# Correlating Optical Reflectance with the Topology of Aluminum Nanocluster Layers Growing on Partially Conjugated Diblock Copolymer Templates

**DOI:** 10.1021/acsami.1c18324

**Published:** 2021-11-17

**Authors:** Marc Gensch, Matthias Schwartzkopf, Calvin J. Brett, Simon J. Schaper, Nian Li, Wei Chen, Suzhe Liang, Jonas Drewes, Oleksandr Polonskyi, Thomas Strunskus, Franz Faupel, Peter Müller-Buschbaum, Stephan V. Roth

**Affiliations:** † Deutsches Elektronen-Synchrotron DESY, Notkestr. 85, 22607 Hamburg, Germany; ‡ Lehrstuhl für Funktionelle Materialien, Physik-Department, Technische Universität München, James-Franck-Str. 1, D-85748 Garching, Germany; § Department of Engineering Mechanics, KTH Royal Institute of Technology, Teknikringen 8, SE-100 44 Stockholm, Sweden; ∥ Wallenberg Wood Science Center, KTH Royal Institute of Technology, Teknikringen 56-58, SE-100 44 Stockholm, Sweden; ⊥ Lehrstuhl für Materialverbunde, Institut für Materialwissenschaft, Christian-Albrechts-Universität zu Kiel, Kaiserstr.2, D-24143 Kiel, Germany; # Gordon Lab, University of California, Santa Barbara, California 93106-5080, United States; ∇ Heinz-Maier-Leibniz Zentrum (MLZ), Technische Universität München, Lichtenbergstraße 1, D-85748 Garching, Germany; ○ Department of Fiber and Polymer Technology, KTH Royal Institute of Technology, Teknikringen 56-58, SE-100 44 Stockholm, Sweden

**Keywords:** polymer−metal interface, optical reflectivity, metal cluster percolation, growth kinetics, diblock copolymer, GISAXS

## Abstract

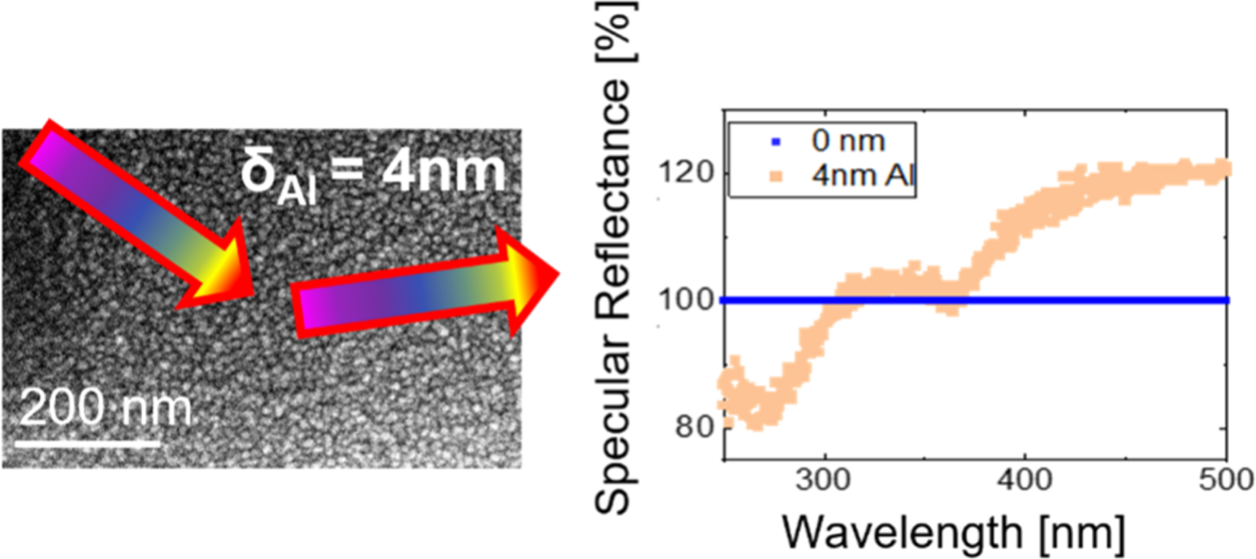

Large-scale fabrication of metal cluster layers for usage in sensor applications and photovoltaics is a huge challenge. Physical vapor deposition offers large-scale fabrication of metal cluster layers on templates and polymer surfaces. In the case of aluminum (Al), only little is known about the formation and interaction of Al clusters during sputter deposition. Complex polymer surface morphologies can tailor the deposited Al cluster layer. Here, a poly(methyl methacrylate)-*block*-poly(3-hexylthiophen-2,5-diyl) (PMMA-*b*-P3HT) diblock copolymer template is used to investigate the nanostructure formation of Al cluster layers on the different polymer domains and to compare it with the respective homopolymers PMMA and P3HT. The optical properties relevant for sensor applications are monitored with ultraviolet-visible (UV-vis) measurements during the sputter deposition. The formation of Al clusters is followed *in situ* with grazing-incidence small-angle X-ray scattering (GISAXS), and the chemical interaction is revealed by X-ray photoelectron spectroscopy (XPS). Furthermore, atomic force microscopy (AFM) and field emission scanning electron microscopy (FESEM) yield topographical information about selective wetting of Al on the P3HT domains and embedding in the PMMA domains in the early stages, followed by four distinct growth stages describing the Al nanostructure formation.

## Introduction

The exploitation of the optoelectronic properties of organic and inorganic nanostructures and cluster layers relies on the ability to direct their self-assembly using chemically or topographically tailored organic and inorganic templates.^1−8^ The utilization of abundant and low-cost metals such as aluminum (Al) is of high interest for surface-enhanced Raman scattering (SERS)-type sensors due to the high absorption in the ultraviolet (UV) spectral range and lower material costs compared to, *e.g*., silver (Ag) or gold (Au).^9−13^ Moreover, thin metal layers of Al or Ag are often used as an electrode material in organic electronics such as in organic photovoltaics (OPVs).^14−19^ A common and facile method to prepare functional metal layers on large scales is sputter deposition.^20−27^ However, the intrinsic physicochemical and nonequilibrium processes during sputter deposition are complex, in particular, when using reactive metals such as Al.^28^ Therefore, a profound view on interaction potentials of the Al with the template material on which sputter deposition takes place is of importance to tailor morphology and optoelectronic properties. For example, Knight et al. investigated the influence of oxidation of Al disks for plasmonic applications and found the tunability of the plasmon resonance frequency in the ultraviolet–visible (UV–vis) spectral range by different amount of oxidation.^29^ Furthermore, Al_2_O_3_ was used as a buffer layer to increase the electrode stability in ambient air.^30−32^ These studies exemplarily show that the chemical interaction of Al with the template needs to be properly understood. To follow the Al layer formation and understand its chemical interaction at polymer surfaces, surface-sensitive techniques with high statistical relevance and more economic viability are important, such as grazing-incidence small-angle X-ray scattering (GISAXS) and X-ray photoelectron spectroscopy (XPS).^33−35^

In previous studies, we investigated the interaction of Ag with polystyrene (PS), poly(methyl methacrylate) (PMMA), and poly(3-hexylthiophen-2,5-diyl) (P3HT) polymer surfaces using surface-sensitive methods such as GISAXS and XPS to reveal the topological changes and chemical interactions.^36,37^ The chemical interaction of noble metals such as Ag was proven with several molecular components, *e.g*., oxygen and sulfur. Ag was reacting with different molecular components of the polymer template as, for example, PS, PMMA, and P3HT, which lead to a different cluster growth depending on the specific interaction.^38^ When moving to less noble metals such as Al, the situation might become even more complex. In earlier work, we investigated the Al formation on P3HT and Alq3 during sputtering in an atmosphere with a high oxygen content,^20,21^ demonstrating the complexity of Al growth on these different organic surfaces. When moving from small molecules or homopolymers to more complex polymer surfaces such as diblock copolymers (DBCs), the complexity increases due to the possibility of having chemically different materials with a distinct nanostructure as the template for the Al deposition. Combining, covalently joint, a conductive, rodlike polymer block such as P3HT with a coil-like polymer block such as PS or PMMA expands the existing knowledge about Al nanostructure formation during sputter deposition.

In the present work, we select PMMA-*b*-P3HT as a template. The focus is on correlating topological changes with optical changes by combining *in situ* UV–vis spectroscopy and GISAXS during Al sputter deposition at the same time. The respective homopolymers PMMA and P3HT are studied for comparison as well. In general, DBC templates are already often used to guide the formation of metal nanoparticles for creating nanostructured hybrid films or to use the self-assembly process on different length scales and functional domains of the DBC to fabricate, *e.g*., optical and medical sensors or as lithography replacement.^39−48^ The specific DBC morphology offers the possibility to improve devices from OPVs and optical sensor applications^25,49^ We monitor the optical response by *in situ* surface differential reflectance spectroscopy (SDRS) and revealed high reduction of the specular reflectance in the UV spectral range until around δ_Al_ = 6 nm for the DBC template. To understand the interaction of Al with the template, XPS measurements are done at early deposition states and show the high affinity of the Al clusters to the molecular components of the DBC. Sputter-deposited Al nanostructures on a PMMA-*b*-P3HT template shows complex nucleation and growth kinetics. The selective wetting of Al on the P3HT block is observed up to an Al thickness of δ_Al_ = 5 nm, and its self-assembly into Al nanostructures is quantified by GISAXS, atomic force microscopy (AFM), UV–vis spectroscopy, and field emission scanning electron microscopy (FESEM). The wetting of Al on the PMMA-*b*-P3HT DBC film shows pronounced growth differences in cluster size, shape, and formation compared to previous studies with Ag on this DBC template.^37^ The direct correlation of morphological, chemical, and optical properties of the Al formation on the DBC gives insight into the early Al–polymer chemical interaction and allows for understanding the formation for large-scale fabrication of optical sensors and electrode materials for organic solar cells.

## Experimental Section

### Materials

One-side polished boron-doped silicon (100) (Si-Mat, Germany) with its native oxide layer was used as substrates (12 × 15 mm^2^ sample size). The polymers poly(3-hexylthiophen-2,5-diyl) (P3HT; molecular weight *M*_n_ = 13.5 kg/mol, polydispersity index, PDI = 1.25), poly(methyl methacrylate) (PMMA; *M*_n_ = 17 kg/mol, PDI = 1.5), and the DBC PMMA-*b*-P3HT, (*M*_n_ = 22-*b*–15 kg/mol, PDI = 2.3, block ratio 1:0.7) (Polymer Source, Inc., Canada) were dissolved in toluene (purity ≥ 99.5%, Carl Roth GmbH, Germany). For sputter deposition, we used a plasma-cleaned 99.999%, 2-inch Al target (Kurt J. Lesker).

### Conjugated DBC Thin-Film Fabrication

The substrates were cleaned for 15 min at 70 °C in the acid solution containing 190 mL of sulfuric acid (96%), 87.5 mL of hydrogen peroxide (30%), and 37.5 mL of ultraclean water (ELGA Purelab Ultra, 18.2 MΩ cm^–1^) to remove all in-/organic residuals.^50^ Afterward, the substrates were cleaned with ultraclean water, dried with nitrogen, and spin-coated (6-RC, SÜSS Micro Tec Lithography, Germany; rpm 3000, ramp 1, time 30 s) with the three different polymer solutions. The concentrations (5 mg/mL) of the polymer solutions for the three different polymer thin films were optimized to have a film thickness δ = (20 ± 3) nm.^37^

### Sputter Deposition

Al sputter deposition on the conjugated DBC thin films was performed in a direct current (DC) magnetron sputter deposition chamber.^23^ The sputter parameters were: power *P* = 110 W, *U* = 286 V, deposition rate *J* = (0.22 ± 0.02) nm/s, base pressure *p*_b_ = 3 × 10^–6^ mbar, and argon flow of *p* = 10 sccm. More details can be found in the Supporting Information.

### Surface Structure Characterization

Details about the AFM, FESEM, and XPS measurements can be found in the Supporting Information.

### Grazing-Incidence Small-Angle X-ray Scattering

For analysis, 10 subsequent two-dimensional (2D) GISAXS data with an exposure time of 0.05 s per frame are summed up to improve statistics. More details about the GISAXS measurements can be found in the Supporting Information.

### *In Situ* UV–vis Surface Differential Reflectance Spectroscopy

Time-resolved UV–vis measurements were performed in reflection mode at a 55° incident angle. Using a deuterium-tungsten halogen light source (DH-2000-BAL, Ocean Insight), the unpolarized light was guided by a fiber optic onto the center of the sample surface. The focused spot size was (0.5 × 0.5) mm^2^. The reflected spectra were transmitted by a fiber optic to a spectrometer (STS-UV, Ocean Insight) in the wavelength range of 250–650 nm and collected every second with an integration time of 200 ms averaged over five measurements. The relative reflectance change was recorded by the ratio between the background-subtracted measured reflectance signal during the Al growth on the polymer sample and the signal of the pristine polymer template. This results in a relative reflectivity change starting at 100% for the pristine polymer template, then increasing reflectivity due to the Al layer deposition, and decreasing reflectivity due to contributions of the plasmon absorption of the Al clusters, the shadowing of pristine substrate reflectivity features and thin-film interference effects. The intensity at λ_1_ = 265 nm and the minima position were extracted by read out in Origin 2020.

## Results and Discussion

### *In Situ* UV–vis, Template-Induced Topography, and Surface Chemistry Characterizations

**Figure 1:**
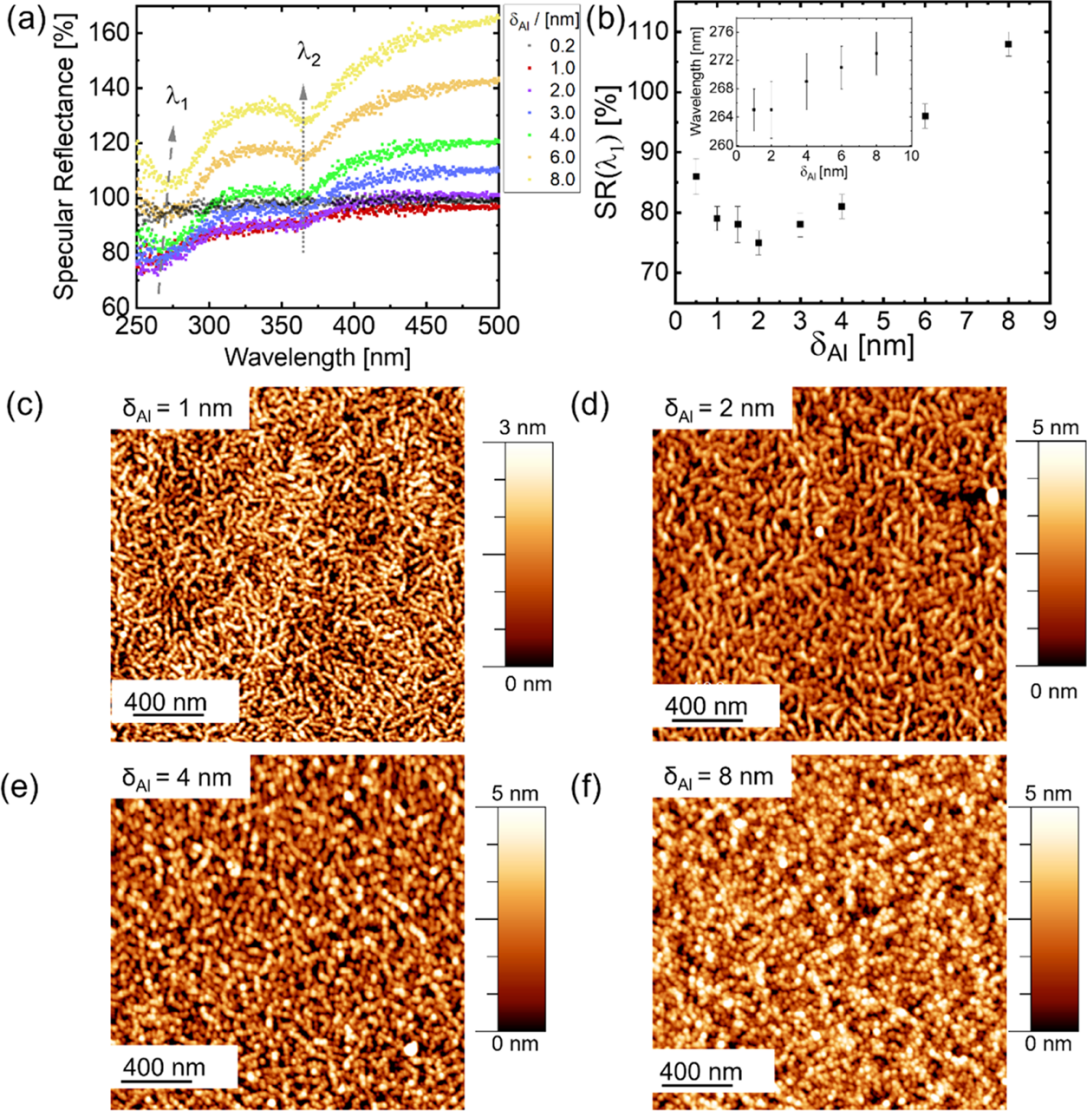
(a) *In situ* UV–vis surface differential specular reflectance data from Al sputter deposition on PMMA-*b*-P3HT, showing two peaks with antireflective behavior in the UV regime. The first local minimum at λ_1_ = 265 nm (dashed gray arrow) and the second local minimum at λ_2_ = 365 nm (dotted gray arrow) from template shadowing effects during Al layer growth are both located in the region of Al plasmonic contributions. (b) Specular reflectance intensity changes at λ_1_ = 265 nm as an indicator of plasmon activity at different effective Al thicknesses reveal a local minimum around δ_Al_ = 2 nm. The inset shows the redshift in λ_1_ minima position. AFM topography of the PMMA-*b*-P3HT film sputter-coated with an Al thickness of (c) δ_Al_ = 1 nm (wormlike Al layer), (d) δ_Al_ = 2 nm (wormlike Al layer), (e) δ_Al_ = 4 nm (nanogranular layer), and (f) δ_Al_ = 8 nm (nanogranular layer).

In the literature, a broad range of Al plasmon absorption from various Al nanoparticles obtained by chemical synthesis or lithographic procedures were reported to be located in the UV–vis spectral region, depending on the size, shape, amount of oxygen, local arrangement, and surrounding medium.^12,29,51−56^ Hitherto, a comprehensive investigation of tuning the morphology and collective optical reflectance of Al layers sputter-deposited on nanostructured DBC thin films is still missing. To highlight the relative changes in the UV–vis reflectance during sputter deposition originating from Al layer growth on the DBC template, the reflectance at an incident angle of 55° of the pristine 20 nm PMMA-*b*-P3HT thin film on a Si wafer is set as reference to 100%. Therefore, observed minima in these surface differential spectra does not directly show the exact position of the localized surface plasmon resonance (LSPR) but provide an indication of an existing plasmon activity as it was shown in our previous publication.^36^ The UV–vis spectra were simultaneously measured with the surface-sensitive X-ray scattering images and thus can be well correlated to the interface morphology of Al growing on the DBC template. For the Al thickness range between δ_Al_ = 1 nm and δ_Al_ = 6 nm, *in situ* UV–vis measurements show a broad region of reduced relative UV reflectivity (Figure 1a), which is located in the spectral region of absorption of Al due to LSPRs. A sequential spectral simulation based on the complex matrix form of the Fresnel equations of a compact (nongranular and nonplasmonic) Al layer growing on top of a 20 nm PMMA/2 nm SiO_2_/Si template reveals that distinct reflection features directly stemming from the template at approximately λ_1_ = (265 ± 3) nm and λ_2_ = (365 ± 2) nm become suppressed by the stratifying Al layers on the topmost interface (see Supporting Information Figure S1). However, the antireflective behavior below λ = 400 nm (relative UV reflectivity <100%) at Al thicknesses below 6 nm is not covered by these simulations, which indirectly signifies the plasmon activity as an additional source of absorption (see the Supporting Information). We assume that, at δ_A1_ = 6 nm, the Al cluster layer reaches its percolation threshold and acts as a rather dense effective medium at which LSPR activity from isolated clusters is drastically decreasing. The specular reflectance in Figure 1b for λ_1_ is reduced to around 78% of the original value of the pristine DBC. This reduced specular reflectance remains below 100% until δ_Al_ = 6 nm. Thus, the localized surface plasmon resonance is changing here to normal surface plasmon absorption, and additional thin-film interference effects become more significant in the optical reflectance. The change of the minima position from the plasmon activity is shown in the inset of Figure 1b. The high and pronounced antireflective specular reflectance in the UV range further shows that such effective Al thicknesses between 1 nm ≤ δ_Al_ ≤ 4 nm are interesting candidates for further investigation of the optical properties by controlling the interface morphology, *e.g*., size, shape, and distance of the Al clusters. There is a need for more comprehensive investigations to obtain profound understanding of thin Al formation by sputter deposition on different polymer templates to prepare well-arranged Al nanostructures for high plasmonic active sensors based on bottom-up procedures.

To follow the formation of the Al cluster layers on the PMMA-*b*-P3HT surface, we perform AFM measurements at selected Al thicknesses. Figure 1c–f shows AFM height images for different Al thicknesses (δ_Al_ = 1, 2, 4, 8 nm). Larger Al thicknesses are shown in Figure S2 in the Supporting Information. A different growth of Al on PMMA-*b*-P3HT is seen compared to Ag on the DBC template, which formed cylindrically shaped Ag clusters on the P3HT domain of the DBC.^37^ At the early stages of the Al sputter deposition (δ_Al_ < 1 nm), Al forms small clusters on the P3HT domain, which connect very fast to wormlike Al nanostructures growing in their size (Figure 1e), comparable to earlier results.^21^Figure 1c (for δ_Al_ = 1 nm) shows wormlike nanostructures on the P3HT domain and small isolated clusters next to the P3HT domains. For δ_Al_ = 2 nm in Figure 1d, the clusters on the P3HT domains are already merged to wormlike nanostructures of Al and cover the entire P3HT domains. The clusters still not appear significantly on the PMMA domains achieving a maximum of selective Al growth induced by the DBC template. The majority of Al clusters are more uniformly distributed and form a nanogranular layer on top of the Al wormlike nanostructures, which induces the higher plasmonic activity around δ_Al_ = 2 nm. We observe a reduced number of well-separated Al clusters compared to the early stages of Ag growth on P3HT homopolymer thin films, while Ag directly forms a nanogranular layer until δ_Al_ = 4 nm.^37^ The wormlike Al growth on the PMMA-*b*-P3HT template is ongoing until around δ_Al_ = 2 nm (as seen in Figure 1d). Subsequently, on the wormlike structures on the P3HT domains, newly forming clusters start to grow, which can be seen at δ_Al_ = 4 nm (Figure 1e). For δ_Al_ = 8 nm in Figure 1f, the Al clusters seem to arrange on the complete surface. This implies that the Al clusters are also emerging more and more on the PMMA domains, and the whole DBC template is fully covered by a percolated Al layer.

**Figure 2:**
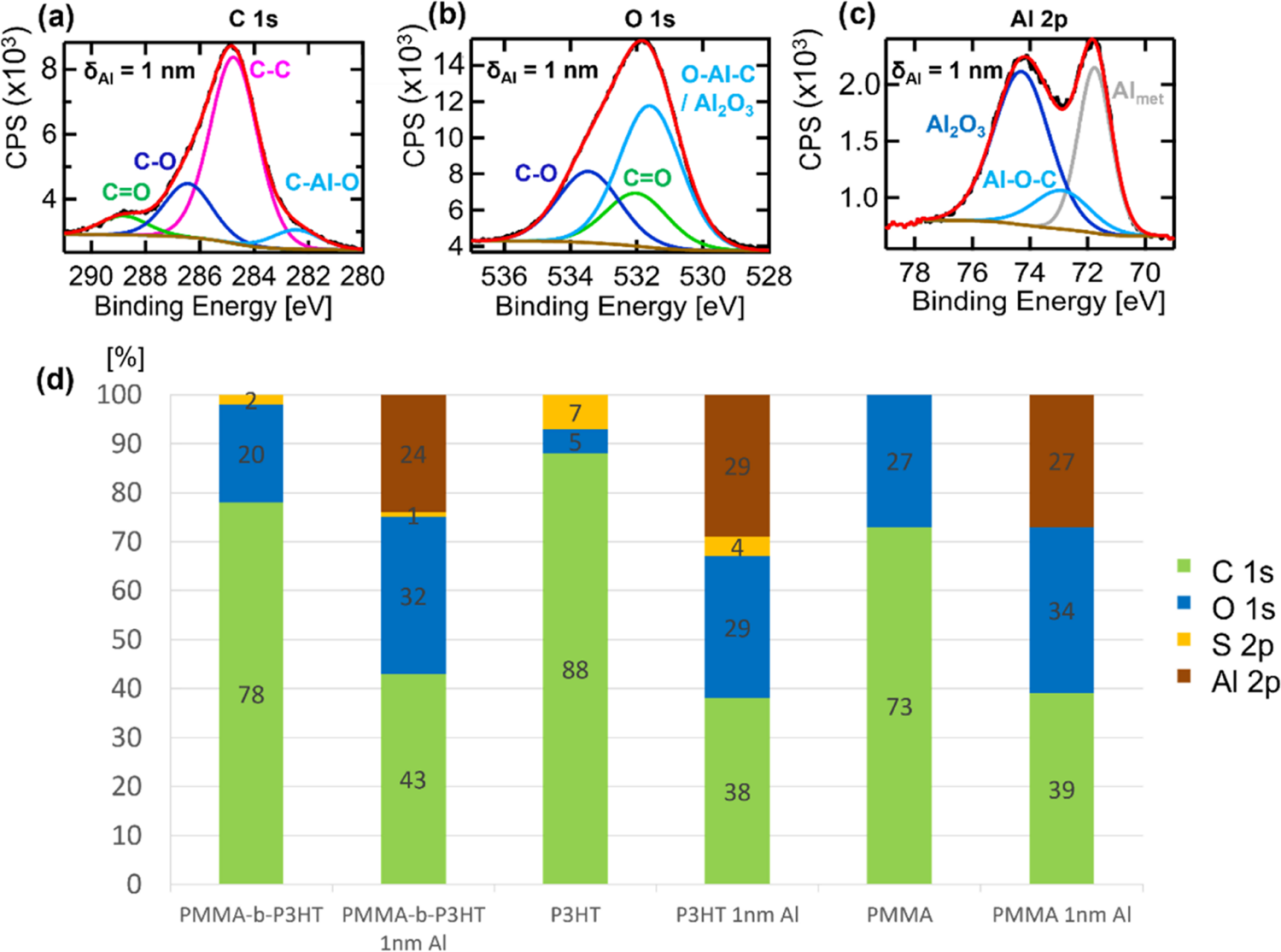
XPS spectra of PMMA-*b*-P3HT with δ_Al_ = 1 nm at (a) C 1s, (b) O 1s, and (c) Al 2p edge. (d) Elemental compositions as the atomic percentage of the corresponding polymer template for δ_Al_ = 0 nm and δ_Al_ = 1 nm derived from the C 1s, O 1s, S 2p, and Al 2p spectra, respectively.

A reason for the different Al cluster growth compared to Ag on the same DBC template^37^ could be the different chemical interaction between the metal and the polymer. This assumption is verified by XPS measurements (Figure 2). Figure 2a shows the C 1s peak of PMMA-*b*-P3HT after deposition of δ_Al_ = 1 nm. A peak is appearing at around 282.5 eV, which is an indication of a C–Al–O compound.^57,58^ A similar interaction with Ag to carbon was not seen for this DBC template in earlier work.^37^ Thus, Al seems to interact with the carbon molecules of the polymer. Furthermore, Figure 2b shows a clear change in the shape of the O 1s peak compared to the pristine DBC sample and to the Ag deposited sample (see Gensch et al.):^37^ The ratio of the intensity of the double peak for the pristine sample changes, which did not change after Ag deposition. The Al 2p peak in Figure 2c arising from the Al formation on the template shows a strong interaction of Al with the polymer domains by the peaks appearing at around 74.3 eV (Al_2_O_3_) and 72.9 eV (Al–O–C). Another peak appears at around 71.8 eV, which shows the proportion of Al metal forming. The high proportion of the oxygen peak in the Al 2p component hints at the early Al cluster formation being highly influenced by the interaction of Al with the molecular components of the polymer domains. This is supported by the wormlike shape of the Al layer on the P3HT domains seen with AFM; the Al and polymer are forming a mixed Al/polymer compound. Zhao et al. showed that for Al nanoparticles, an Al_2_O_3_ shell formed around the Al nanoparticles.^59^ Such oxide shell might be a reason for the fast formation of a wormlike mixed Al/polymer layer because the flat Al clusters with an Al_2_O_3_ shell are connecting fast and interacting strongly with the molecules of the P3HT domain for the first two nanometers of Al deposited. The forming Al_2_O_3_ compositions are seen in Figure S3 for the DBC template and the respective homopolymers P3HT and PMMA. Bou *et al*. showed that the metal peak, as seen in Figure 2c, increased with further Al deposition, whereas the oxygen peak in the Al 2p spectra was negligible for thicker Al thickness, which might enhance therefore the plasmon response.^57,58,60^ The same tendency of metallic Al is seen for the Al interaction in the case of the homopolymers P3HT and PMMA (Figure S4) for the C 1s and O 1s peaks. After Al deposition, the O 1s peak in the data of the P3HT sample indicates a high amount of Al_2_O_3_, which might result from the interactions with oxygen in the surrounding atmosphere (Figure S6). The appearing oxygen compound peak shows the high interaction potential of Al with the molecular components of the polymer and with oxygen (Figures 2b and S6). For Al, the interaction with the components is highly dependent on the oxygen content in the polymer, which can be seen in Figures S3, S4c, and S4f (Supporting Information). The relation between the Al metal peak and Al oxygen peak in the Al 2p spectra is depending on the reactive molecules in the polymer template, as it can be seen in Figures S4c and S4f in the Supporting Information. Here, the Al interaction peak with oxygen and carbon depends on the oxygen amount and molecular arrangement in the polymer. Figure 2d compares the atomic proportions of the molecular components with δ_Al_ = 0 nm to δ_Al_ = 1 nm. The overall area for the C 1s peak is decreasing with Al deposition, which could be related to the bonding of Al with carbon molecules on the polymer template, in agreement with the appearance of the O–Al–C peak and the decreasing of the C=O peak. For the O 1s peak, the overall proportions are increasing after Al deposition. This trend is explained by the addition of two components at the O=C peak position after Al deposition. Here, the signals of Al_2_O_3_ and O–Al–C are overlapping with the O=C component. This indeed shows the bonding of Al with the oxygen and carbon molecules of the polymer template. The information related to the specific compound percentage for all templates with and without Al is detailed in Figures S3, S5, and S6 in the Supporting Information. The S 2p peak for sulfur did not show significant changes before and after Al deposition for the DBC and only intensity reduction after deposition (mostly at the S–H compound) for the P3HT homopolymer and the DBC, as shown in Figures S7 and S8 (SI). The reduction of the intensity can be an indication for the formation of the mixed Al/polymer layer for the DBC template. The Al might interact at the S–H group with the sulfur to metal sulfide, but no significant peak is seen after deposition, in contrast to that observed for Ag before.^37^ This fits well to the chemistry of these two metals; while Ag has a high affinity to sulfur and a much smaller affinity to oxygen, the opposite holds for Al. Here, a strong bonding is expected to oxygen sites, while the bonding to sulfur should be weak.

### Selective Wetting Analysis

**Figure 3:**
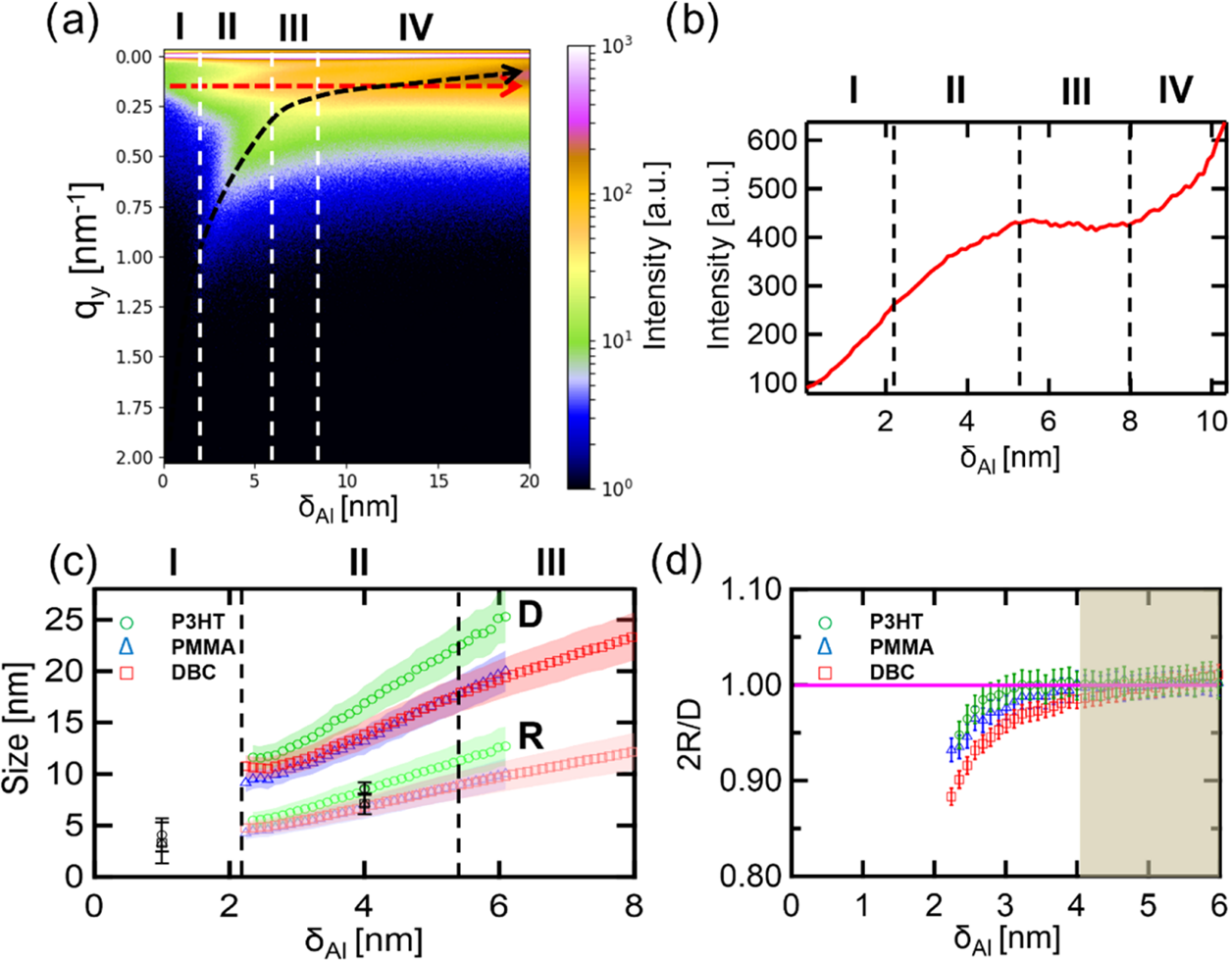
(a) Contour plot of horizontal line-cuts from the *in situ* GISAXS data measured during sputter deposition on PMMA-*b*-P3HT as a function of effective Al thickness δ_Al_. The position of the cluster peak (black dashed arrow) is changing due to the Al deposition and the position of the first-order DBC domain peak (red dashed arrow) at *q*_*y*_ = 0.15 nm^–1^ is constant during Al deposition. The white dashed vertical lines indicate different growth regions as explained in the text. (b) Amplitude of the domain peak of PMMA-*b*-P3HT during Al sputter deposition. (c) Average Al interparticle distance on PMMA (*D*_PMMA_, blue), PMMA-*b*-P3HT (*D*_PMMA-*b*-P3HT_, red), P3HT (*D*_P3HT_, green), and the corresponding cluster radii for the different polymer films *R*_PMMA_ (bright blue), *R*_PMMA-*b*-P3HT_ (bright red), and *R*_P3HT_ (bright green). The radii calculated from FESEM images at δ_Al_ = 1 and 4 nm are shown as black symbols. (d) Ratio of the average cluster diameter (2*R*) and the mean interparticle distance (*D*) ratio for PMMA (blue), PMMA-*b*-P3HT (red), and P3HT (green). When 2*R*/*D* = 1 (magenta line), the clusters start to touch each other, resulting in a macroscopic conductive path. The brown box covers the region, in which the Al reaches the percolation on the three templates.

Figure 3a shows the contour plot of the horizontal line-cuts from the *in situ* GISAXS data at the Yoneda peak position (Figure S9). The domain peak of the DBC is indicated by a red arrow in Figure 3a, while the cluster peak is shown by a black arrow. The different growth regimes are separated with white dashed lines and indicated by Roman numbers: (I) nucleation, embedding, the point of first observation of the cluster peak and formation of the merged cluster layer on P3HT; (II) selective diffusion-mediated cluster growth *via* coalescence and cluster growth on the Al merged cluster layer; (III) reduced-selective adsorption-mediated growth and percolated regime on the P3HT domain; (IV) percolated layers on both polymer domains. In Figure 3b, the selective wetting indicated by an increasing scattered intensity induced by an increased electron density contrast is followed by the intensity evolution of the domain peak of the DBC. A similar effect was found for the sputter deposition of Ag on the same template.^37^ Selective wetting can be seen until around δ_Al_ ≈ 2 nm in Figure 3b. At this point, the Al cluster growth starts on the merged Al layer on P3HT and on PMMA. Afterward, the intensity increase on the P3HT domain slows down, which can be a hint for the reduced selectivity. The maximum at δ_Al_ = 5.2 nm indicates the following decrease in the selective wetting. Al starts to cover more and more surface on both domains. At around δ_Al_ ≈ 8 nm, the cluster peak is starting to overlap with the DBC domain peak, which leads to an increase in its intensity and is indicative of the connecting, merged Al cluster layer on both polymer domains. Figure 3c shows the cluster radii (*R*) and the mean center-to-center distances (*D*) of Al on PMMA-*b*-P3HT (red), PMMA (blue), and P3HT (green). The radii are derived from the geometrical model using the film thickness and the center-to-center distances assuming a hemispherical cluster shape as introduced in an earlier study.^14^ The growth on all templates shows a linear growth with a constant slope for *R* and *D* (in contrast, Ag showed different slopes in the different growth regions),^37^ which we take as a hint for the reduced diffusion of atoms on the polymer surface. The increased chemical interaction of Al with the polymer domains leads to a fast bonding of Al to the polymer, thereby slowing down the diffusion of the Al clusters/atoms on the DBC domains (PMMA and P3HT).

**Figure 4:**
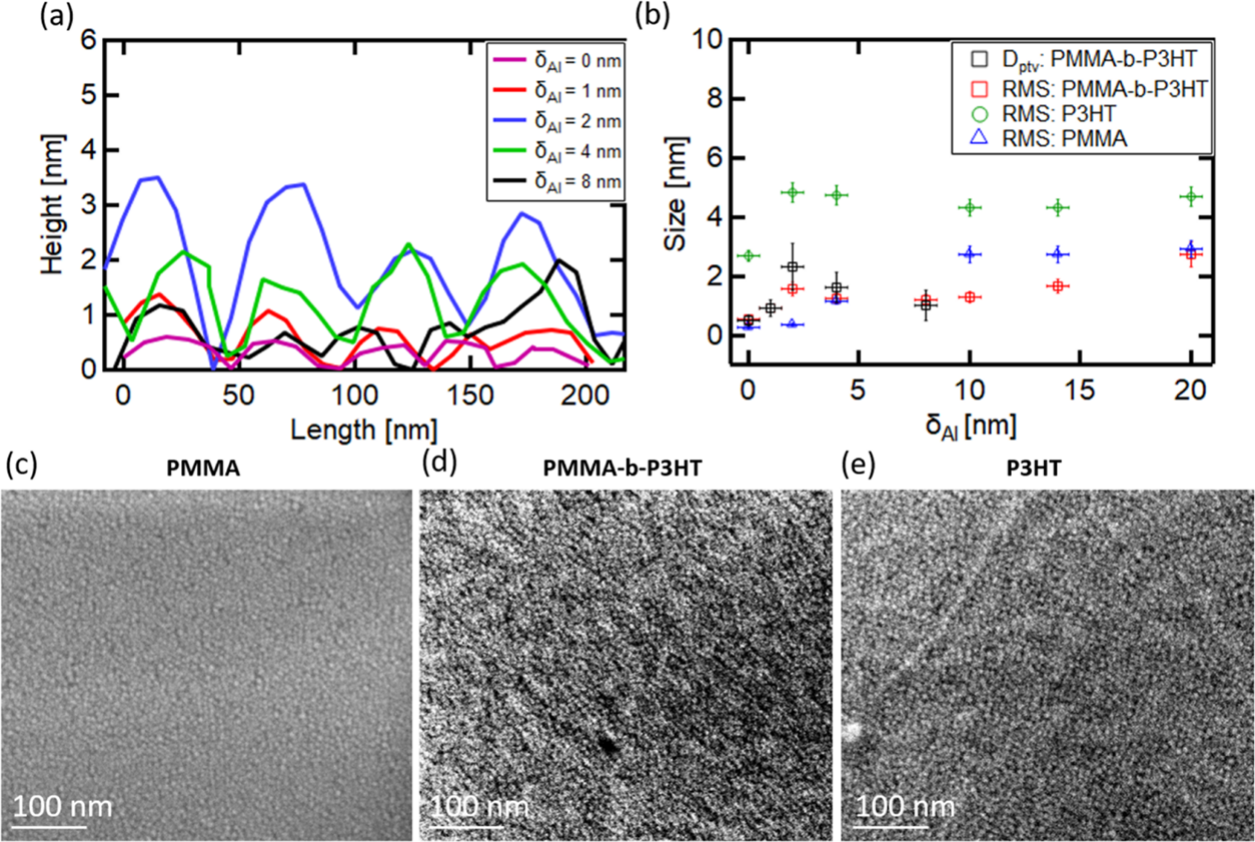
(a) Line-cut from AFM data of PMMA-*b*-P3HT films sputter-coated with a different Al thickness as indicated. (b) Average peak-to-valley distance (*D*_ptv_) and root-mean-square (RMS) roughness measured with AFM for different Al thicknesses on PMMA-*b*-P3HT, PMMA, and P3HT. FESEM images for (c) Al/PMMA, (d) Al/PMMA-*b*-P3HT, and (e) Al/P3HT with δ_Al_ = 1 nm. A homogeneous Al cluster distribution is observed on all templates.

**Figure 5:**
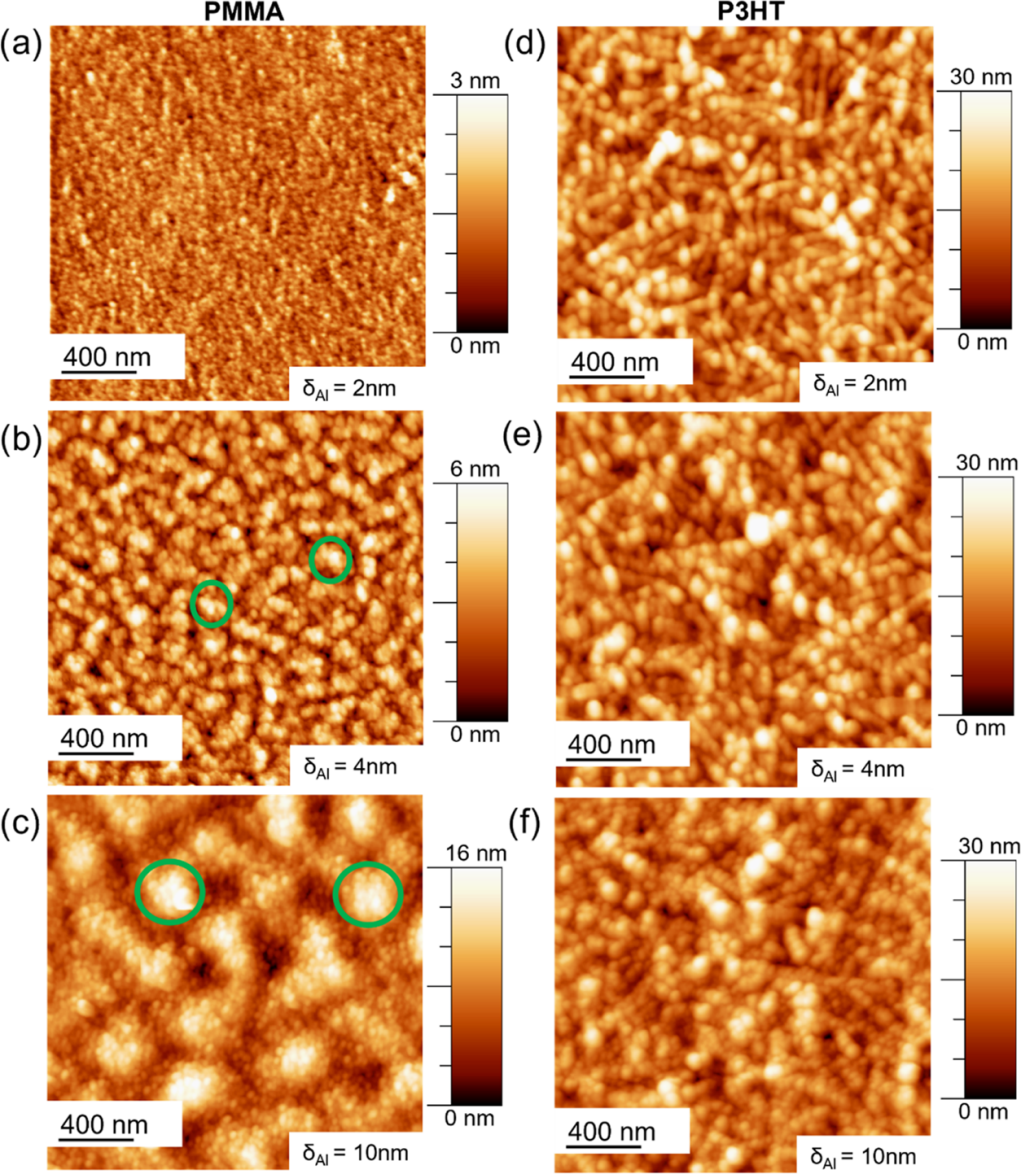
AFM topography of (a–c) PMMA and (d–f) P3HT thin films sputter-coated with Al thicknesses of (a, d) δ_Al_ = 2 nm, (b, e) δ_Al_ = 4 nm, and (c, f) δ_Al_ = 10 nm. Green circles show the cluster aggregation regions of Al on PMMA.

Compared to typical Au or Ag growth on polymer thin films, the interaction of Al with the polymer is higher.^22,36,37^ The increased chemical interaction is seen by the high interaction peak in the Al 2p spectra (Al_2_O_3_ and O–Al–C compounds) and is consistent with the bonding, which could be considered as defects on the polymer thin films and could limit the diffusion of the metal atoms on the films. When correlating the growth with FESEM results (Figures 4c–e and S10a–S10c) in Figure 3c (black markers), which reveal nearly the same cluster radii for δ_Al_ = 4 nm, a linear growth is found for the radii and center-to-center distances for both homopolymers P3HT and PMMA. However, on P3HT, larger Al clusters are seen compared to PMMA (as also seen by AFM and FESEM) above δ_Al_ ≈ 3 nm. In the case of the DBC, the center-to-center distances show a deviation in the slope from a linear growth (Figure 3c), which is related to the different Al growth on the polymer domains of the DBC and therefore resulting in a later percolation. In the region of δ_Al_ = 5–6 nm, the linear increase for the center-to-center distances slows down, which indicates the transition from the selective diffusion-mediated cluster growth *via* coalescence on the merged cluster layer on P3HT to the reduced-selective adsorption-mediated growth and percolated regime on the P3HT domain. In Figure 3d, the deduced percolation analysis is shown, which is calculated from the geometrical model.^22^ The percolation threshold is not as clearly developed as for Ag on these templates. A region (brown box) can be identified where Al percolates at δ_Al_ = 4–6 nm, which is in excellent agreement with the FESEM data (Figures S10a–S10c). Overall, the Al growth on the DBC template is smoother and more uniform due to a more controlled growth on the more ordered domains on the DBC template compared to the randomly oriented fibers of the P3HT homopolymer (see Figure 5d) or on the PMMA template with different cluster aggregation regions (see Figure 5b). Therefore, the DBC template has a sharper percolation transition between δ_Al_ = 5–6 nm compared to the respective homopolymers, which would be advantageous for electrodes. An observable peak in the GISAXS data of the PMMA sample located at smaller *q*_y_ values for the aggregation regions (second cluster peak in Figure S9j) is not considered in the calculations for the percolation.

The growth of Al on the DBC domains is studied *ex situ* in more detail in Figure 4. Figure 4a shows line-cuts from the AFM data for different Al thicknesses to reveal the selective wetting of the Al merged cluster layer on the PMMA-*b*-P3HT template. The change in the average peak-to-valley distance (*D*_ptv_) in comparison to the pristine substrate is an indicator for the selective wetting of the metal on one polymer domain, similar to Ag.^37^ In Figure 4b, the selective wetting is found at around δ_Al_ = 2 nm from the analysis of the line-cuts, where *D*_ptv_ reaches a maximum. The difference compared to Ag is the fast-forming wormlike nanostructure of Al (see Figure 1c,d). A merged cluster layer on the P3HT domain forms quickly already at δ_Al_ = 1 nm until δ_Al_ = 2 nm and then changes to a cluster growth again on top of the wormlike nanostructure. At the same time, the embedding and subsurface growth of Al on PMMA (see Figure 5a,b and the increasing RMS value in Figure 4b) are reduced, and the growth extends to the surface of PMMA. In contrast, when depositing Ag on this template, Ag atoms self-assemble solely in clusters without forming such a merged cluster layer. Above δ_Al_ = 2 nm, the selective decoration of Al is still present for Al clusters on the merged Al cluster layer until around δ_Al_ = 8 nm but is reduced as seen in Figure 4b by the reduction of the growth increase of *D*_ptv_. *D*_ptv_ is following the overall root-mean-square (RMS) roughness of the DBC thin film and is visible until δ_Al_ = 8 nm, which shows the end of the selective wetting on the DBC template by the Al clusters. Afterward, the Al clusters fill up the valleys of the PMMA domains and the value of *D*_ptv_ decreases significantly. The merged cluster layer formation and smooth Al cluster growth on the DBC template are also confirmed by the analysis of line-cuts on the P3HT domain of the DBC, seen in Figure S11. The Al clusters and the merged cluster layer underneath do not show a change in the height distribution until δ_Al_ = 8 nm (see Figure S11 in the Supporting Information). This finding could be an indication for the smooth merged cluster layer formation in the beginning, followed by a homogeneous cluster arrangement on the Al merged cluster layer. Figure 4b confirms such a scenario, where the RMS roughness values of the Al growth on the homopolymers are compared to the RMS roughness values on the DBC, which is lower until δ_Al_ = 20 nm. Hence, the Al layer formation on the DBC seems to be much smoother compared to the Al layer formation on the respective homopolymers. However, the RMS roughness value increases significantly for the DBC compared to the homopolymers in the later stages of the sputter deposition.

The FESEM images in Figure 4c–e confirm the uniform cluster growth independent of the polymer template in the early stages up to δ_Al_ = 4 nm (Figure S10a–c). In Figure 4c (PMMA), the contrast is lower compared to the DBC and P3HT in Figure 4d,e. The lower contrast can be a hint for the embedding of the Al clusters inside the polymer film, as it can be seen also by AFM measurements (Figure 5a, and RMS roughness values for PMMA in Figure 4b). In Figure 4d, the merged cluster layer seems to arrange on the DBC on the P3HT domains, in agreement with the AFM images (Figure 1c,d). In contrast to the early stages (δ_Ag_ < 2 nm) of Ag cluster growth on this DBC template, we do not observe Al cluster aggregation regions on the crystalline part of the P3HT domains. Instead, a homogeneous distribution on the P3HT is visible. On the P3HT thin film (Figure 4e), the Al clusters are homogeneously distributed and seem to be slightly larger compared to the other templates (DBC and PMMA), as also seen by the AFM measurements (Figure 5).

To improve the understanding of the Al cluster formation on the DBC, the Al growth on the respective homopolymers is analyzed in more detail. In Figure 5a–c, AFM height images of the Al growth on PMMA are shown at selected Al thicknesses. Al shows a similar embedding behavior as previously reported for Ag on PMMA.^36^ The Al clusters embed and start a subsurface growth for the first 2 nm of deposited Al, as indicated by the low RMS roughness value for δ_Al_ = 2 nm on PMMA (Figure 4b). In Figure 5b, Al clusters are visible together with some aggregation regions (green circles). These regions can be seen in more detail at a larger Al film thickness, *e.g*., for δ_Al_ = 10 nm (Figure 5c). For comparison, in Figure 5d–f, AFM topography images of the Al growth on P3HT are shown. Figure 5d shows the Al merged cluster layer, replicating the homopolymer fiber structure of the pristine P3HT thin film. In Figure S12 in the Supporting Information, the pristine films of P3HT (fibers), PMMA (homogenous thin polymer film), and the DBC template (P3HT domain around PMMA standing cylinders)^37^ can be seen. In Figure 5d, it seems that the Al atoms adsorbed from the vapor phase on the P3HT domain are chemically interacting directly with the P3HT domain and are forming clusters homogeneously arranged on the fibers. This can be further seen in Figure 5e,f, where the clusters continuously grow laterally. For δ_Al_ > 10 nm, a uniformly distributed cluster layer is observed (Figure S13). The identification of sputtering metallic Al is found in the GIWAXS measurements. The ring-shaped intensity distribution (Figure S14) results from a powder texture of the Al grains. The position of the intensity rings corresponds to the (111) and (200) plane of metallic Al.

### Growth Model

**Figure 6:**
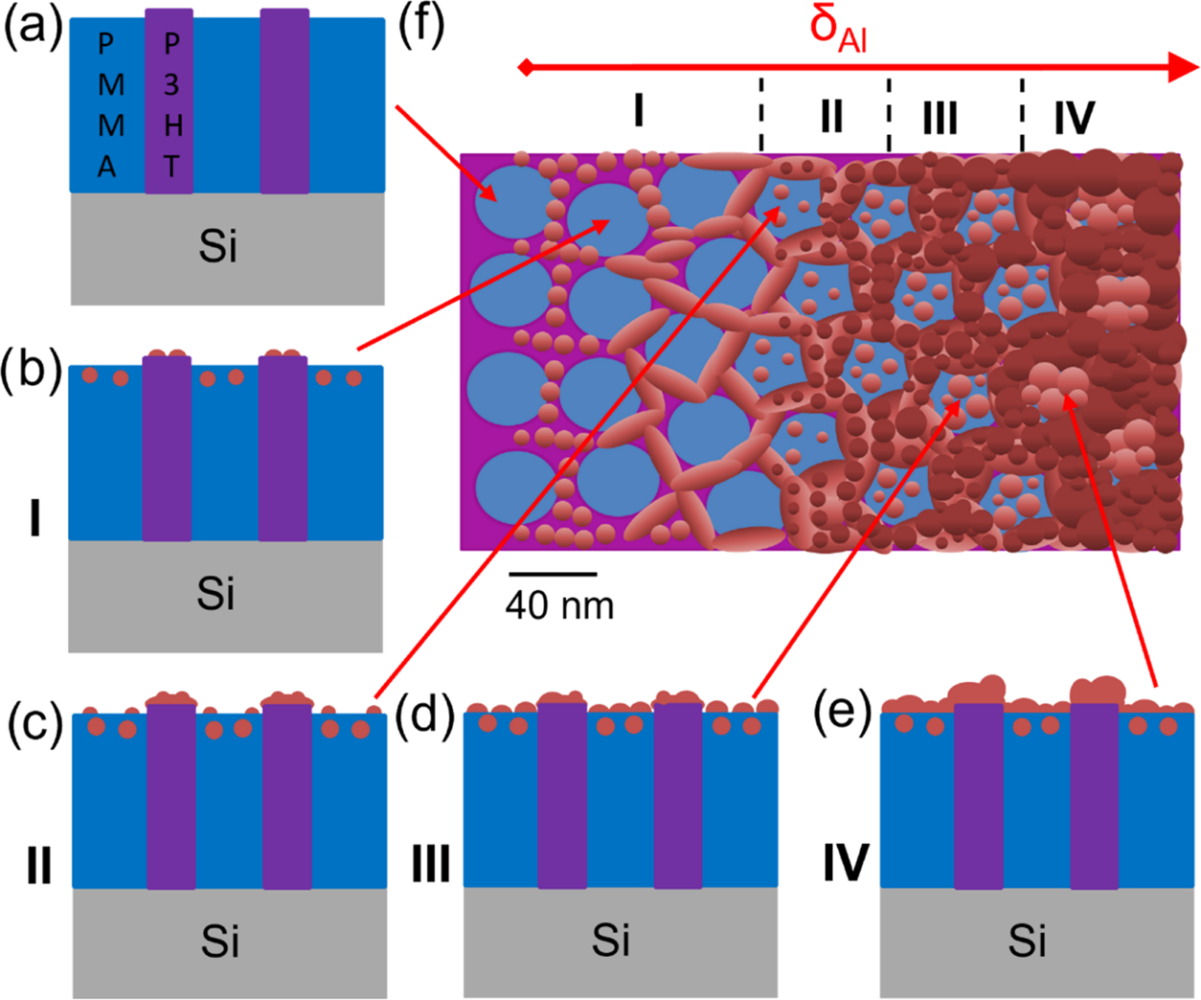
Sketch of the growth model of Al (brown clusters) on the PMMA-*b*-P3HT DBC template in (a–e) side view and (f) from the top. Roman numbers I–IV indicate different growth regimes as described in the text.

Combining all results, it is possible to derive a model for the different growth regimes, as shown in Figure 6. Figure 6a–e sketches of the side view of the pristine and Al-deposited DBC together with the established DBC morphology.^37^ The nucleation, embedding, and subsurface growth are shown in Figure 6b, regime (I). Figure 6c shows the selective diffusion-mediated cluster growth *via* coalescence on the merged cluster layer on P3HT, which defines the next regime (II). The reduced-selective adsorption-mediated growth and percolated regime on the P3HT domains are shown in Figure 6d, regime (III). The percolated layers on both polymer domains are sketched in Figure 6e and mark the final regime (IV). A top view of all growth regimes is sketched in Figure 6f with the flat Al clusters on the P3HT domains at the beginning of deposition, which are then merged to the wormlike Al layers on the P3HT domains (merged cluster layer). Subsequently, newly adsorbed Al atoms form well-ordered Al clusters on the merged Al layers. After embedding and subsurface growth on the PMMA domains (I), the Al atoms form continuously growing Al clusters on the PMMA surface (II and III). The Al clusters on the Al merged Al layer continuously grow in size, as seen also on the fibers of the P3HT homopolymer thin film, to a nanogranular Al layer (III).

## Conclusions

The optical properties and the complex growth of Al nanostructures in four growth stages on a PMMA-*b*-P3HT DBC film are quantified exploiting *in situ* methods. A difference in the cluster arrangement on PMMA is found compared to the other polymer templates P3HT and PMMA-*b*-P3HT. We find that the early Al growth on P3HT follows a fast percolation of clusters to a merged cluster layer on the P3HT fibers and domains. On PMMA, first, embedding of Al in the polymer is observed. Then, clusters start to grow to a rough cluster layer. The XPS measurements show the high chemical interaction of Al with the molecular components of the polymers and even for the P3HT domain to form Al_2_O_3_ or Al–O–C compounds, which could in turn affect plasmon activity. We correlate the UV–vis *in situ* relative reflectance change of the pristine DBC template to the change with Al decoration and observe an antireflective behavior in the UV regime for Al thicknesses below the percolation threshold, which can be attributed to Al plasmon resonance. Thus, a clear correlation between the morphology of nanogranular Al merged cluster layers to its optical and morphological properties is presented. Such a direct correlation is of interest because of the increased high absorption of ≈20% for UV light and due to the achieved homogeneous Al cluster distribution: The plasmon activity in 2 nm thin Al films is important for sensor applications and could be used with these metal–polymer hybrid films to fabricate optical sensors. Thus, these results will impact the tailoring of optically active metal merged cluster layers for sensors and could be also expanded to organic photovoltaic applications when using polymer-assisted sputter deposition.

## Supporting Information Available

Further details on UV–vis, GISAXS, GIWAXS, AFM, and FESEM images and XPS measurements (PDF)
