# Supplementary material for: Correlating Optical Reflectance with the Topology of Aluminum Nanocluster Layers Growing on Partially Conjugated Diblock Copolymer Templates
Source: ACS Appl Mater Interfaces. 2021 Nov 17;13(47):56663–73. doi: 10.1021/acsami.1c18324 (PMC8640968; doi:10.1021/acsami.1c18324)
Supplement: Supplementary file 1 — am1c18324_si_001.pdf [file am1c18324_si_001.pdf]

SUPPORTING INFORMATION

Correlating Optical Reflectance with Topology of Aluminum Nanocluster Layers Growing on Partially Conjugated Diblock Copolymer Templates

Marc Gensch,†,§, Matthias Schwartzkopf,†, Calvin J. Brett#,†,@, Simon J. Schaper§, Nian Li§, Wei Chen§, Suzhe Liang§, Jonas Drewes•, Oleksandr Polonskyi~, Thomas Strunskus•, Franz Faupel•, Peter Müller-Buschbaum§,% and Stephan V. Roth*,†,&*

† Deutsches Elektronen-Synchrotron DESY, Notkestr. 85, 22607 Hamburg, Germany

§ Lehrstuhl für Funktionelle Materialien, Physik-Department, Technische Universität München, James-Franck-Str. 1, D-85748 Garching, Germany

Department of Engineering Mechanics, KTH Royal Institute of Technology, Teknikringen 8, SE-100 44 Stockholm, Sweden

@ Wallenberg Wood Science Center, KTH Royal Institute of Technology, Teknikringen 56-58, SE-100 44 Stockholm, Sweden

• Lehrstuhl für Materialverbunde, Institut für Materialwissenschaft, Christian-Albrechts-Universität zu Kiel, Kaiserstr.2, D-24143 Kiel, Germany

~ Gordon Lab, University of California, Santa Barbara CA 93106-5080, USA

% Heinz-Maier-Leibniz Zentrum (MLZ), Technische Universität München, Lichtenbergstraße 1, D-85748 Garching, Germany

& Department of Fiber and Polymer Technology, KTH Royal Institute of Technology, Teknikringen 56-58, SE-100 44 Stockholm, Sweden

* Corresponding author

marc.gensch@desy.de

stephan.roth@desy.de; svroth@kth.se

KEYWORDS

polymer-metal interface; optical reflectivity; metal cluster percolation; growth kinetics; diblock copolymer; GISAXS

Outline

1) UV-Vis simulations and spectra
2) Sputter deposition
3) Atomic Force Microscopy
4) Field Emission Scanning Electron Microscopy
5) X-ray Photoelectron Spectroscopy
6) Grazing-Incidence Small- and Wide- Angle X-ray Scattering

1) UV-Vis Spectroscopy

Simulations of SDRS spectra based on the complex-matrix form of the Fresnel equations of a growing compact (non-granular and non-plasmonic) Al layer on top of 20 nm PMMA/2nm SiO2/Si substrate at different Al thickness (from 0 to 20 nm) using Reflectance Calculator from Filmetrics® supports interpretation of experimental SDRS data (Figure S1). The calculated reflectance spectra are normalized to a simulated PMMA (20nm) thin film on a SiO_2(2nm)/Si-substrate (violet curve). The obtained spectra sequence in the range of 200 nm to 500 nm quantifies the evolution of optical reflectance features solely due to changes in the thin-film interference conditions in the UV-Vis regime. The antireflective behavior for Al thickness below 4nm (percolation threshold) is not covered by the simulations and yields probably indirectly the plasmon activity as an additional source of absorption below 300 nm. This fits very well to the extracted GISAXS percolation threshold. LSPR gets lost when metallic clusters percolating to a metallic layer. At the higher Al

thicknesses well above 6 nm, the SDRS intensity ratio agrees very well to the simulations. This indicates the Al layers fully stratifies as an optically effective medium on top of the template, which is primarily modulating the UV-Vis light path and optical reflectance is obeying the Fresnel equations.

Formula for the measured specular reflectance (SR):

$$\mathrm{SR} = \frac{\boldsymbol{recorded\ spectra\ during\ sputter\ deposition - dark\ spectra}}{\boldsymbol{pristine\ polymer\ spectra - dark\ spectra}} \times \mathbf{100\%} \qquad (1)$$

2) Sputter deposition

The effective metal thickness of the sample was calculated using a quartz crystal microbalance (QCM, Inficon, Switzerland), which was positioned above the sample before and after the deposition, thus acting also as a shutter system. For the XPS measurements, the same DC sputter source and QCM system were used and connected to the XPS chamber (Kiel University, CAU, Institut für Materialwissenschaft). More details about the sputter chamber can be found in the publication of Schwartzkopf *et al.*.[1]

3) Atomic Force Microscopy

NSG03 semi-contact cantilevers with a tip radius of (6 nm) and a resonant frequency of (90 ± 8) kHz (NT-MDT, Russia) were used to measure AFM. The program Gwyddion (v2.53) was used to analyze the AFM data and the program WSXM (v5.0) from Horcas *et al.* was used to visualize the data.[2,3] The line-cuts shown in Figure S5 are averaged line-cuts from 3 line-cuts in

the original AFM images. The line-cuts have a resolution of one data point per 7.8 nm in the lateral direction. The sputtered samples for the AFM images were prepared by the DC sputter chamber.

4) Field Emission Scanning Electron Microscopy

FESEM images were obtained with a Zeiss Ultra Plus (Carl Zeiss SMT, Germany) at an aperture size of 10 µm with an accelerating voltage of 3 kV. The high voltage 3 kV was used for the aluminum coated samples to avoid accumulation of charges. A secondary electron in-lens detector was used to measure the secondary electron signal in beam direction without backscattering electrons. The sputtered samples for the FESEM images were prepared by the DC sputter chamber.

5) X-ray Photoelectron Spectroscopy

X-ray photoelectron spectroscopy (XPS, Omicron Nano-Technology GmbH, Germany) was applied to investigate the chemical environment of sputter deposited aluminum clusters on the different polymer templates (P3HT, PMMA, and PMMA-*b*-P3HT) before and directly after 1 nm Al sputter deposition as described above. The above-mentioned DC sputter chamber was directly connected to the XPS device at the University of Kiel (CAU, Institut für Materialwissenschaft). The XPS was operated with an Al anode at a power of 240 W and spectra were taken with a VSW 100 hemispherical analyzer with pass energies of 100 eV (survey) and 30 eV (high-resolution spectra). CasaXPS software (version 2.3.19, www.casaxps.com) was used to charge reference the spectra using the carbon C 1s at 284.8 eV to quantify the amount and chemical shifts of carbonyl moieties before and after aluminum deposition.

6) Grazing-Incidence Small- and Wide- Angle X-ray Scattering

An incident photon energy of 13 keV was used with a beam size of (31 × 24) μm^2 at the sample position. The sample-to-detector distance (SDD) was set at SDD_1 = (2462 ± 2) mm for *in situ* GISAXS using a Pilatus 300K (Dectris Ltd., Switzerland; pixel size of (172 × 172) μm^2) at the beamline P03 (DESY, Hamburg).[4] The direct transmitted and specular reflected beams were both shielded by two separate beam stops to avoid saturation or damage to the detector. In order to achieve a good separation between the polymer and aluminum Yoneda peaks, an incident angle of $\alpha_i = 0.39°$ was selected during the *in situ* GISAXS measurements. In order to prove macroscopic homogeneity of the nanoscale morphology and to avoid possible X-ray beam effects during the *in situ* GISAXS experiments, the entire sputter chamber was laterally moved over two-millimeter distances during the experiment using a goniometer (HUBER Diffraktionstechnik GmbH & Co. KG, Germany). Meanwhile, the scattering data were continuously recorded at a frame rate of 20 images per second for GISAXS. The GISAXS data was analyzed using the DPDAK software package[5]. The sample-to-detector distance for *in situ* GIWAXS was set at SDD_2 = (178 ± 3) mm using a LAMBDA 750k detector (X-Spectrum GmbH, Germany; pixel size of 55 × 55 μm^2) at the beamline P03 (DESY, Hamburg).

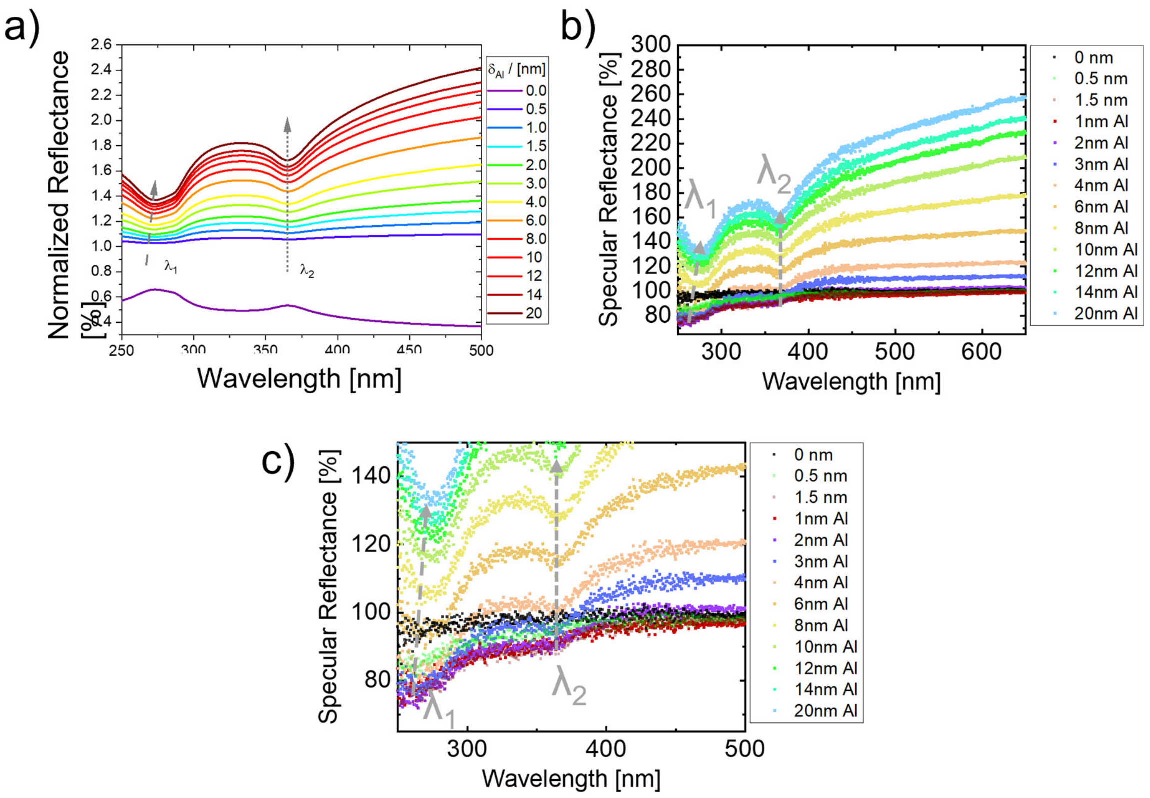

Figure S1: a) Simulations of SDRS spectra based on the complex-matrix form of the Fresnel equations of a growing compact (non-granular and non-plasmonic) Al layer on top of 20 nm PMMA/2nm SiO2/Si substrate at different Al thickness (from 0 to 20 nm). Reflection features λ_1 (dashed grey arrow) and λ_2 (grey dotted arrow) appear at same positions of the template (violet spectra for 0 nm Al) and become pronounced by stratifying the different Al layers on top. b) Specular reflectance full spectra from δ_{Al} = 1 nm to 20 nm. c) Zoom in UV-region of the specular reflectance from b).

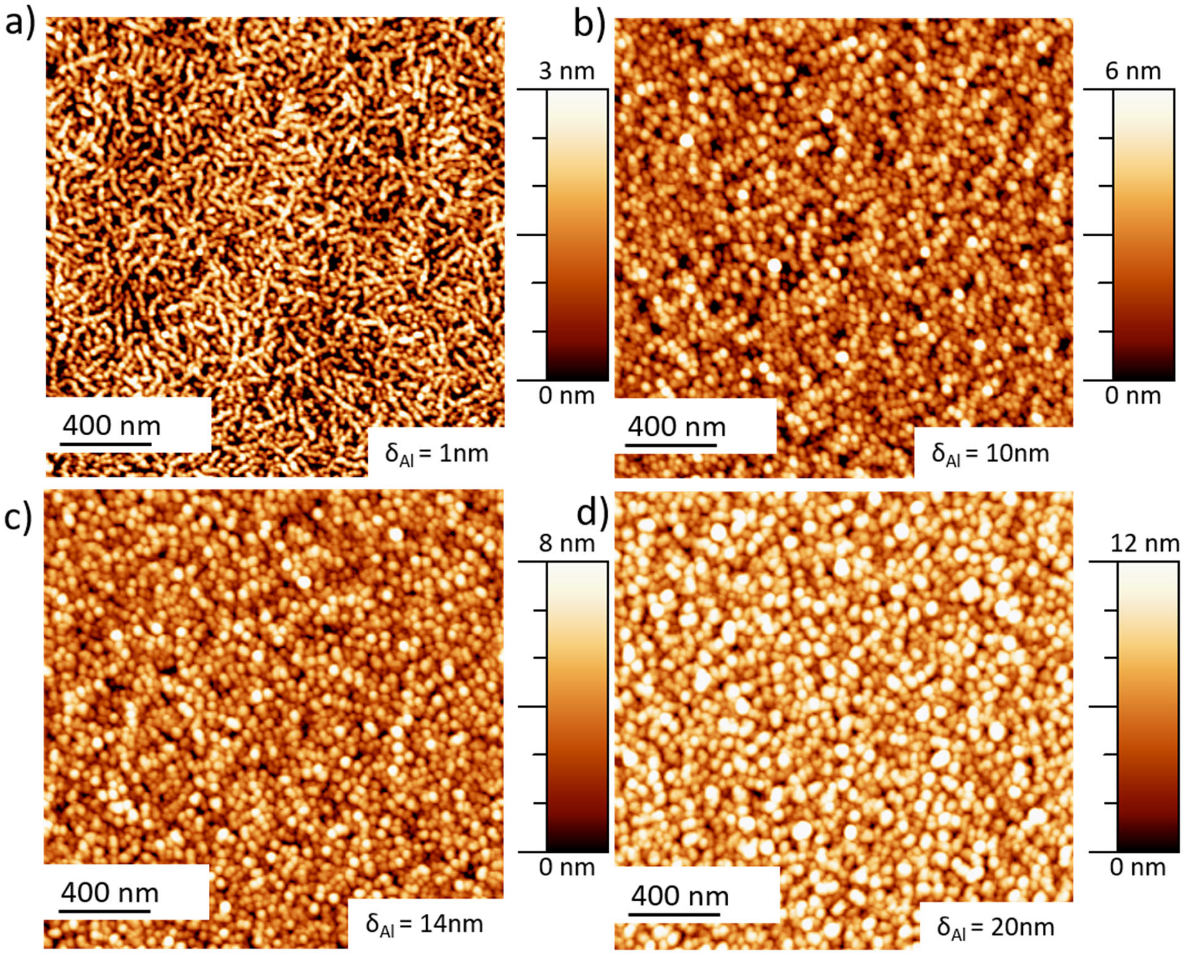

Figure S2: AFM height images of PMMA-*b*-P3HT films with sputter deposition of δ_{Al} = a) 1 nm, b) 10 nm, c) 14 nm and d) 20 nm.

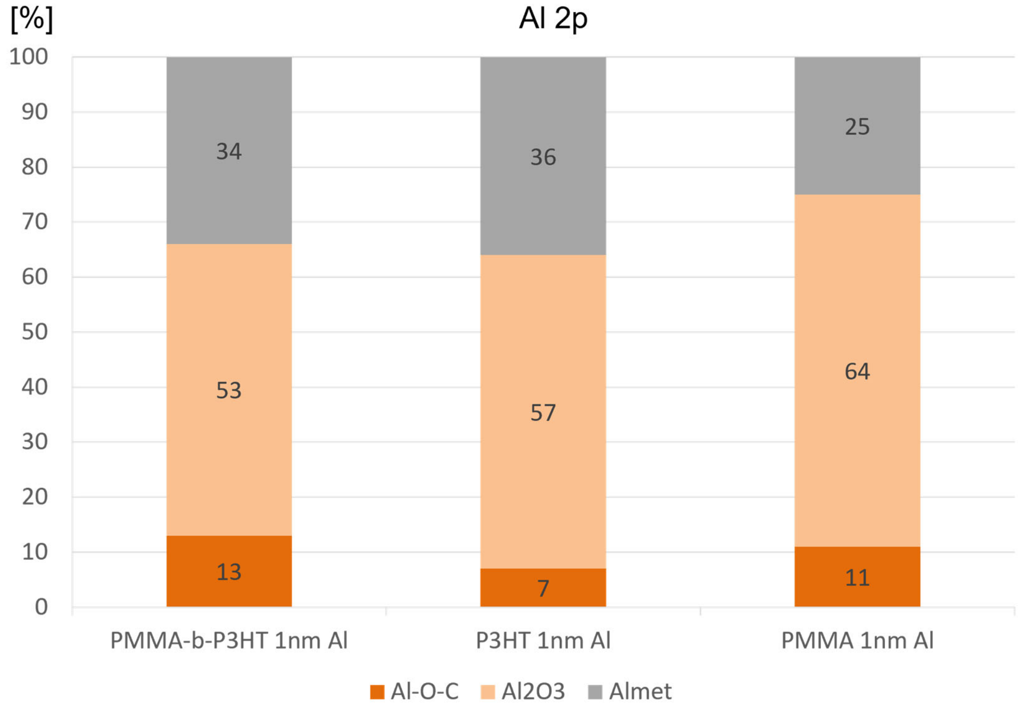

Figure S3: Contributions to the XPS spectra measured at the Al 2p edge of PMMA, P3HT and PMMA-*b*-P3HT films after sputter deposition of δ_{Al} = 1 nm.

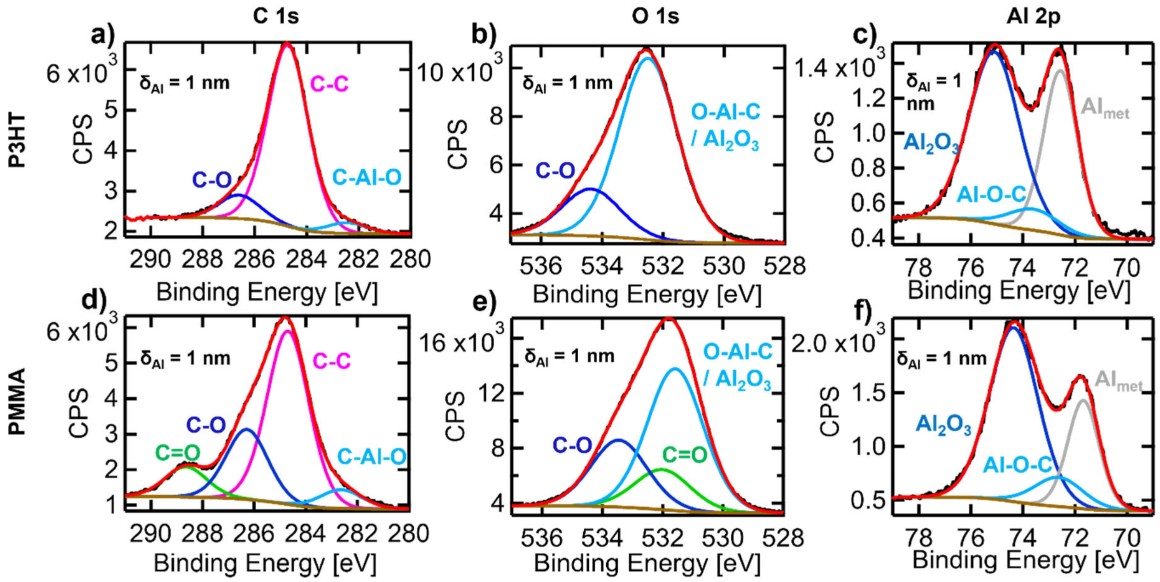

Figure S4: XPS spectra at C 1s, O 1s and Al 2p edge of **a), b), c)** P3HT, **d), e), f)** PMMA films after sputter deposition of δ_{Al} = 1 nm.

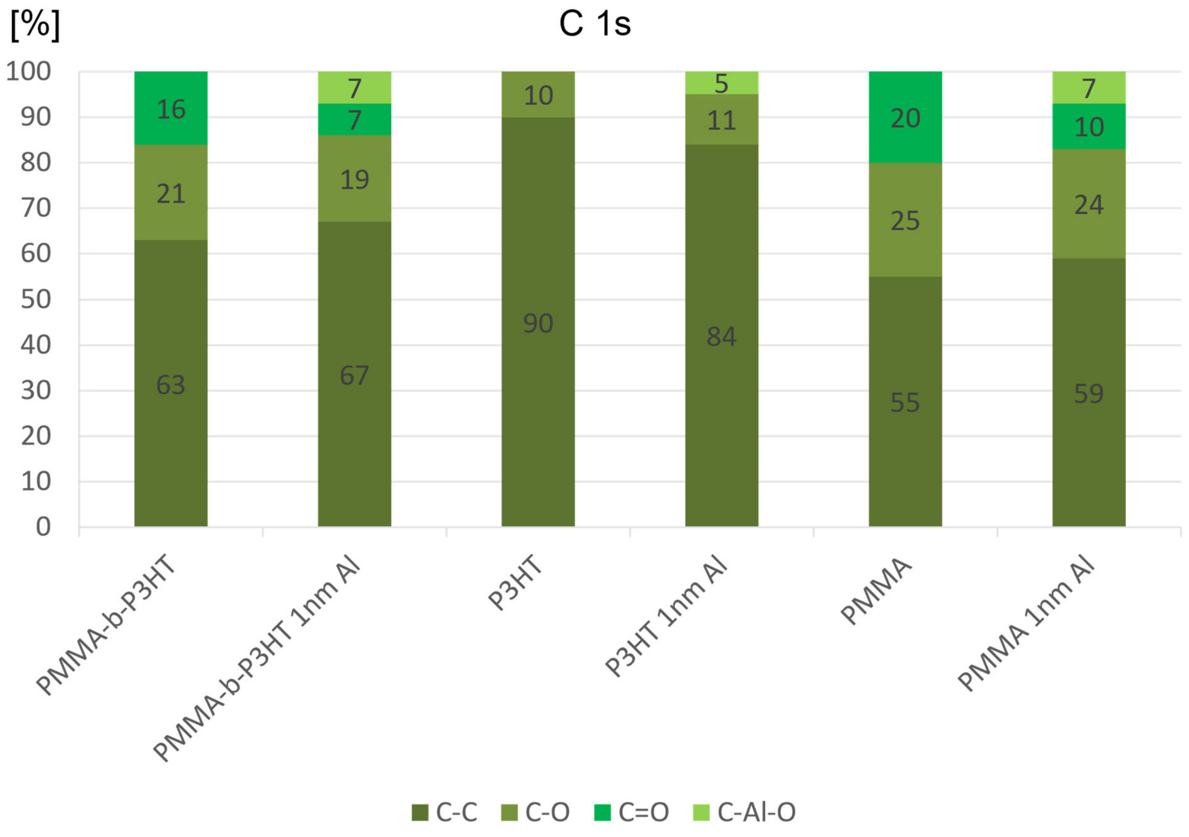

Figure S5: Contributions to the XPS spectra measured at the C 1s edge of PMMA, P3HT and PMMA-b-P3HT films before and after sputter deposition of $\delta_{Al} = 1$ nm.

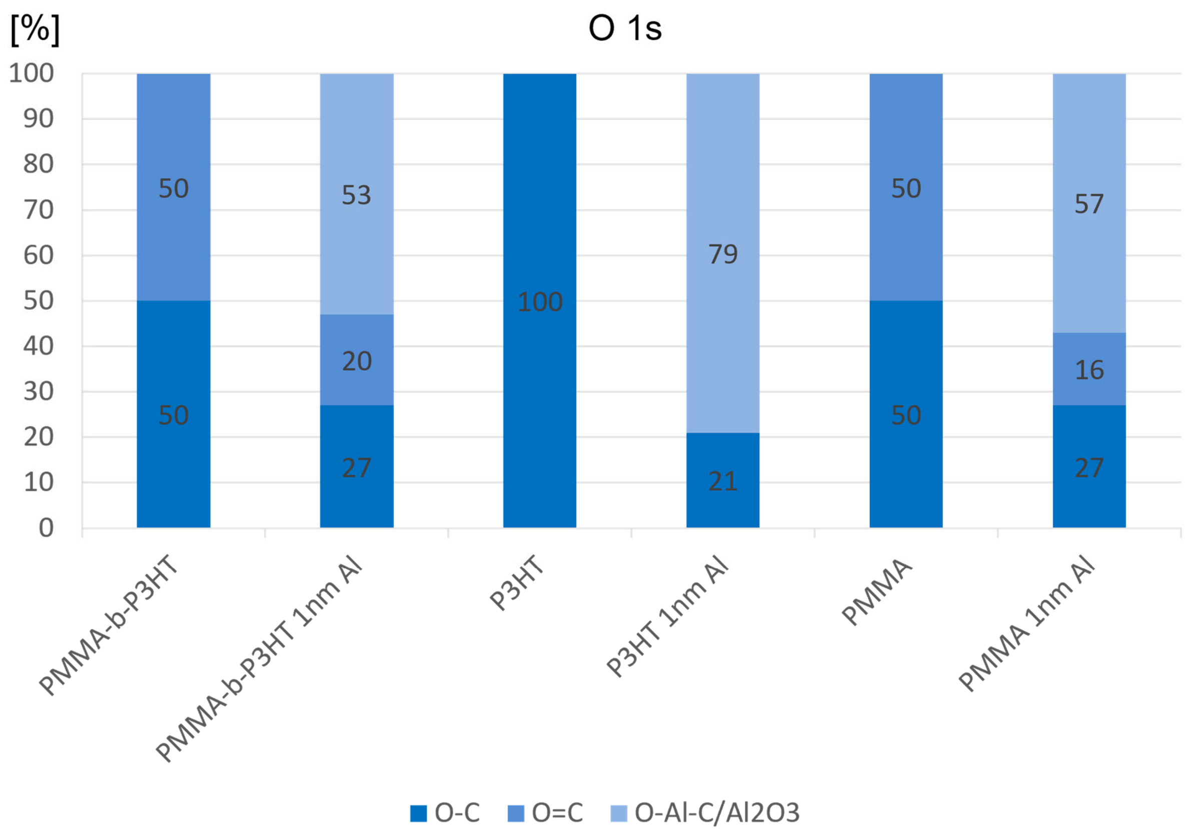

Figure S6: Contributions to the XPS spectra measured at the O 1s edge of PMMA, P3HT and PMMA-b-P3HT films before and after sputter deposition of δ_{Al} = 1 nm.

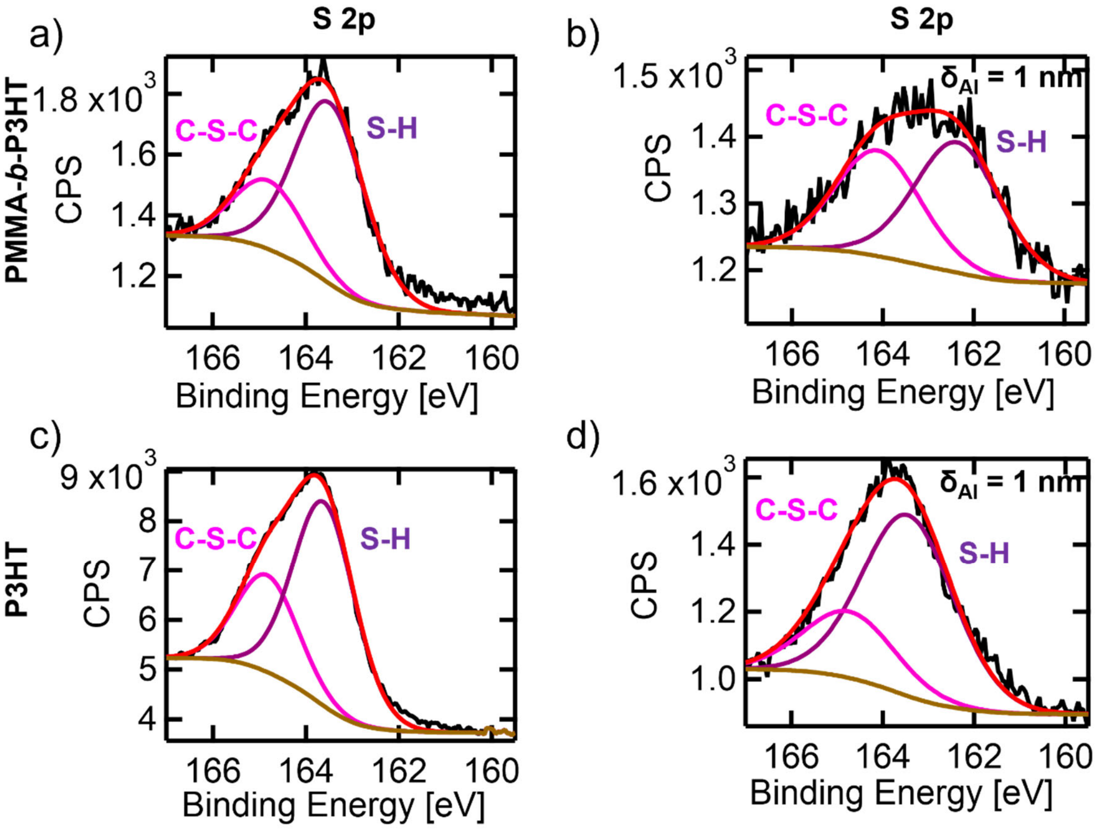

Figure S7: XPS spectra at the S 2p edge of **a,b)** PMMA-*b*-P3HT and **c,d)** P3HT films before and after sputter deposition of δ_{Al} = 1 nm.

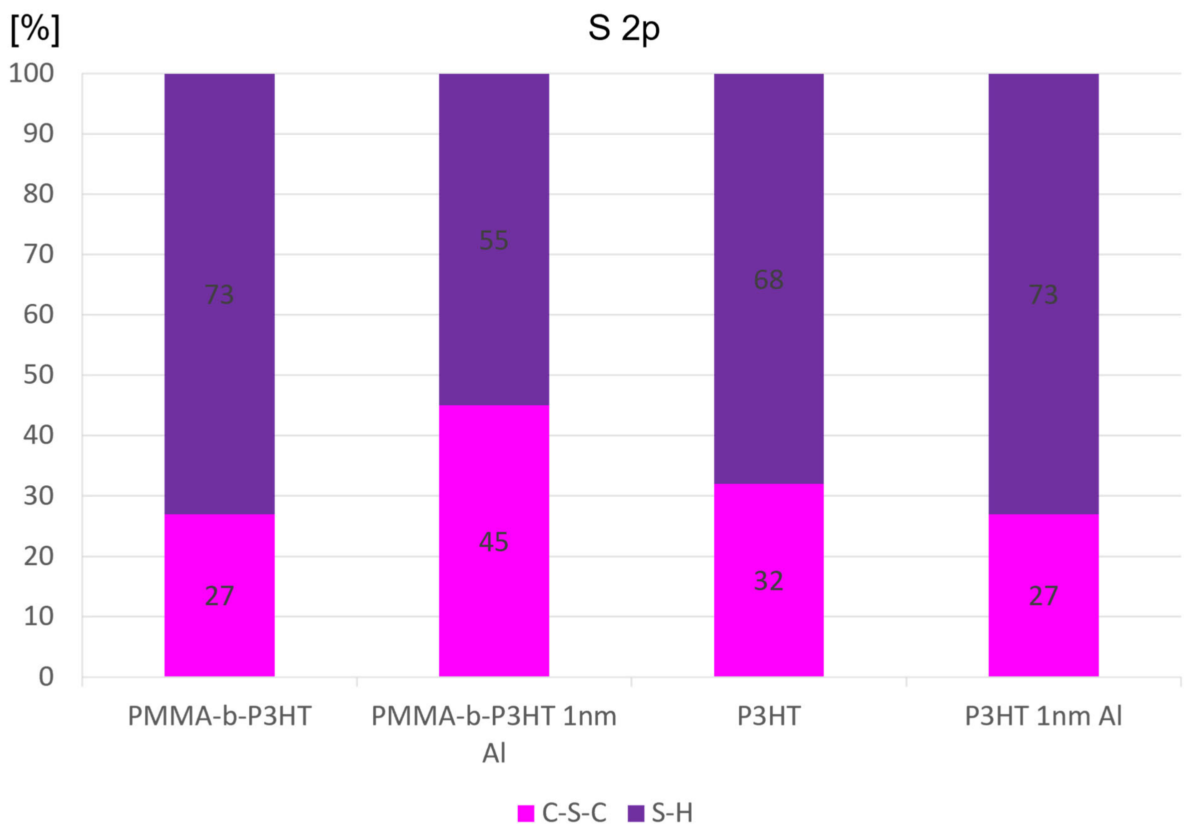

Figure S8: Contributions to the XPS spectra measured at the S 2p edge of P3HT and PMMA-b-P3HT films before and after sputter deposition of δ_{Al} = 1 nm.

The Al layer formation on the polymer films is shown in the 2D GISAXS data in Figure S8. The homopolymer thin films are compared with the diblock copolymer for different Al thicknesses (δ_{Al} = 2 nm, 5nm, 10 nm and 14 nm). The low electron density of Al makes it challenging to detect the Al nanostructures in the X-ray experiment, especially for very low Al thicknesses. The Al cluster peak and the diblock copolymer domain peak are indicated by white arrows in Figure S8a. The cluster peak moves to smaller q_y positions in the lateral direction, which corresponds to a growth in the size and center-to-center distance of the clusters. The GISAXS data and FESEM images give information about the cluster shape, which we assume to be a half sphere as first order approximation for all studied polymer templates in this work.[6] From the GISAXS pattern, the shape

of the cluster peak seems to be the same for all templates. This is corroborated by FESEM measurements (Figure S9) leading to a similar percolation threshold in the region of around δ_{Al} = 5-6 nm comparing very well to the percolation threshold determined in Fig. 3d by GISAXS. This finding is different compared to earlier work about Ag on these polymer templates, where we found a cylindrical cluster shape on the crystalline part of the of the P3HT both, in the homopolymer thin film and the P3HT domains of the DBC, where the crystalline part acts as a defect for the Ag atoms.[7]

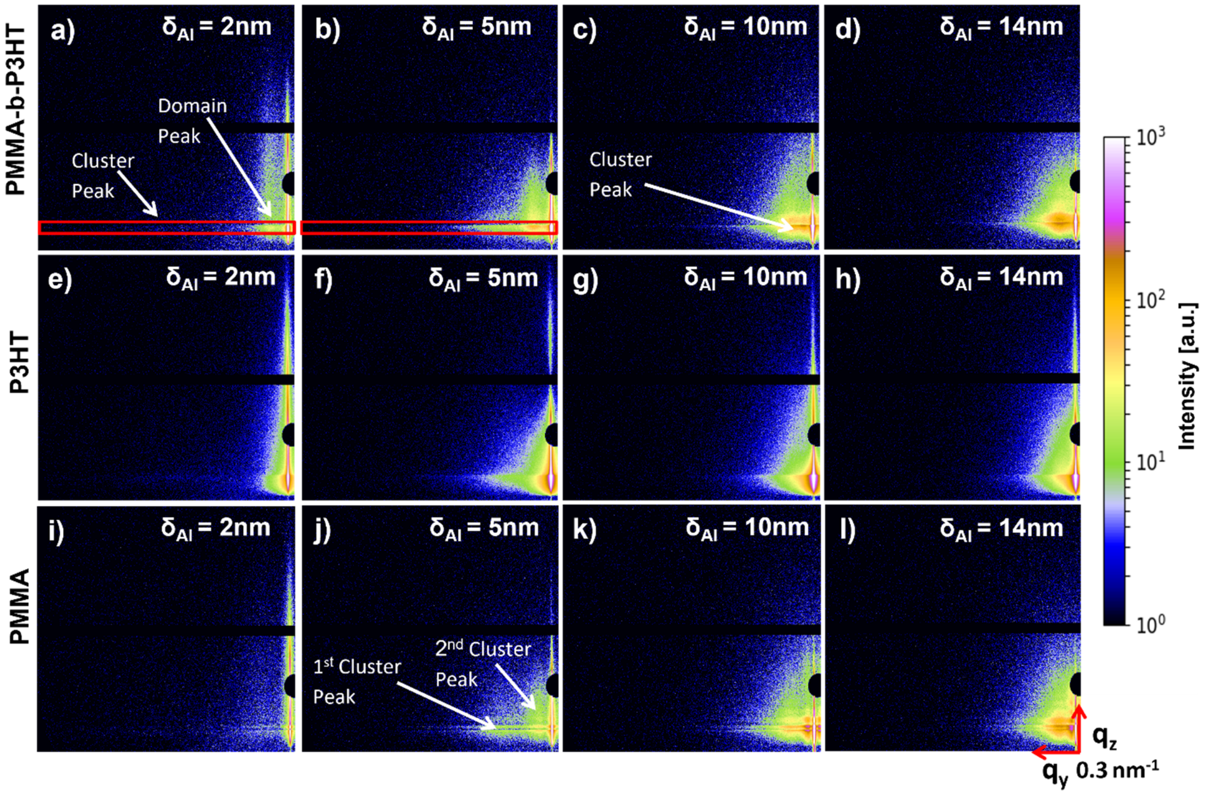

Figure S9: Selected 2D GISAXS data of Al cluster growth on **a-d)** PMMA-*b*-P3HT, **e-h)** P3HT, and **i-l)** PMMA for Al thicknesses of δ_{Al} = 2 nm, 5 nm, 10 nm and 14 nm, respectively. The red boxes show the Yoneda region for the lateral line cuts. The domain peak and cluster peaks are highlighted. The scattering pattern indicate a hemispherical shape of the Al nanostructures for all templates.

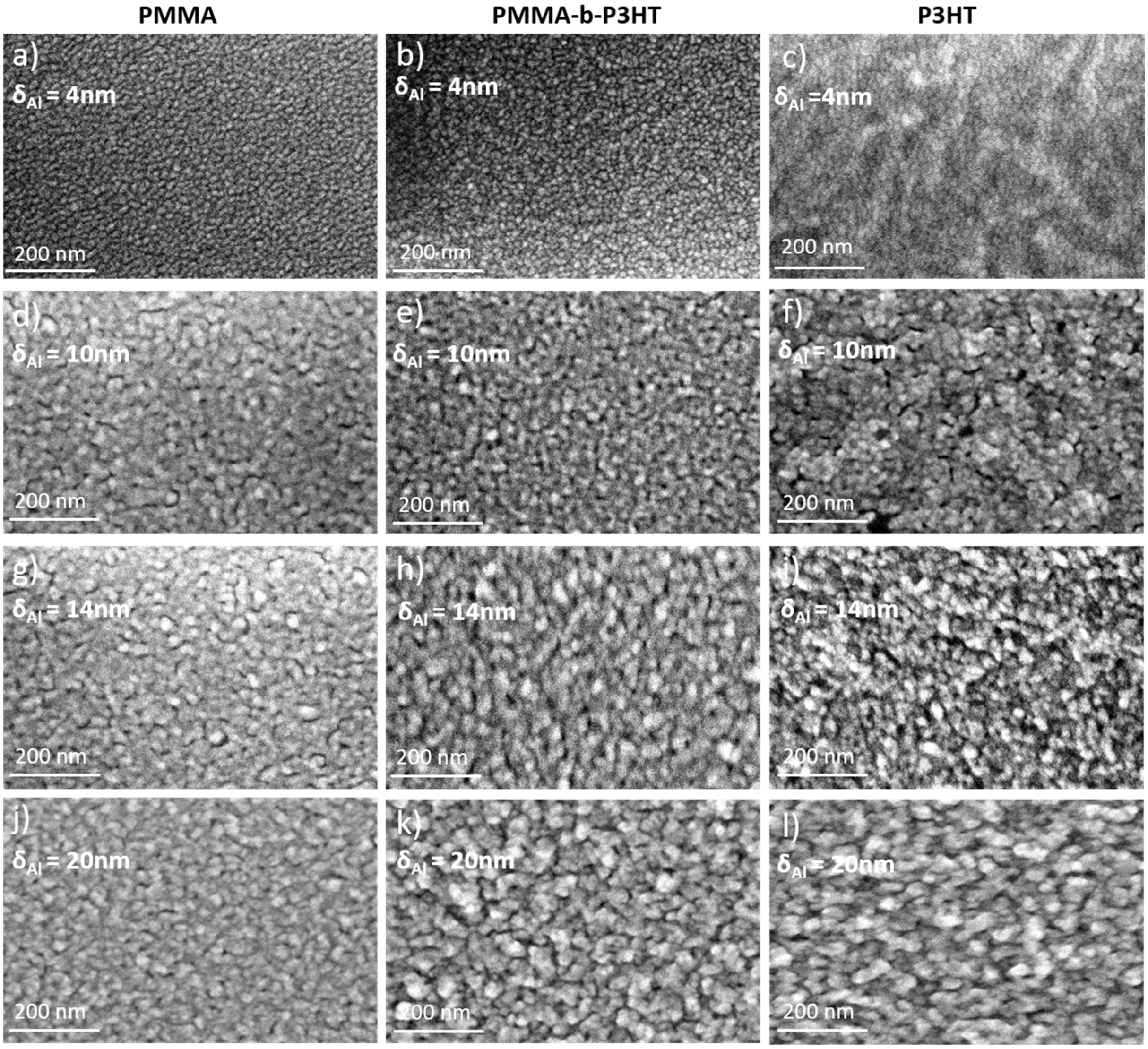

Figure S10: FESEM images **a,d,g,j)** PMMA, **b,e,h,k)** PMMA-*b*-P3HT, and **c,f,i,l)** P3HT with δ_{Al} = 4 nm, 10 nm, 14 nm and 20 nm.

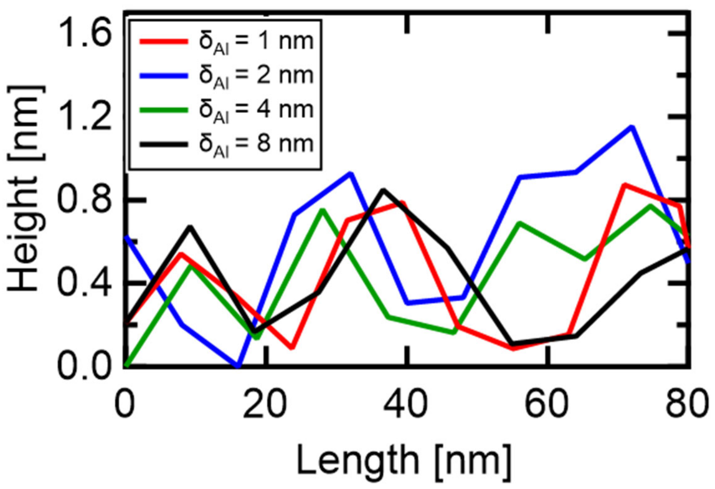

Figure S11: Line-cut from AFM data on the P3HT domain of PMMA-*b*-P3HT films sputter coated with different Al thicknesses as indicated**.**

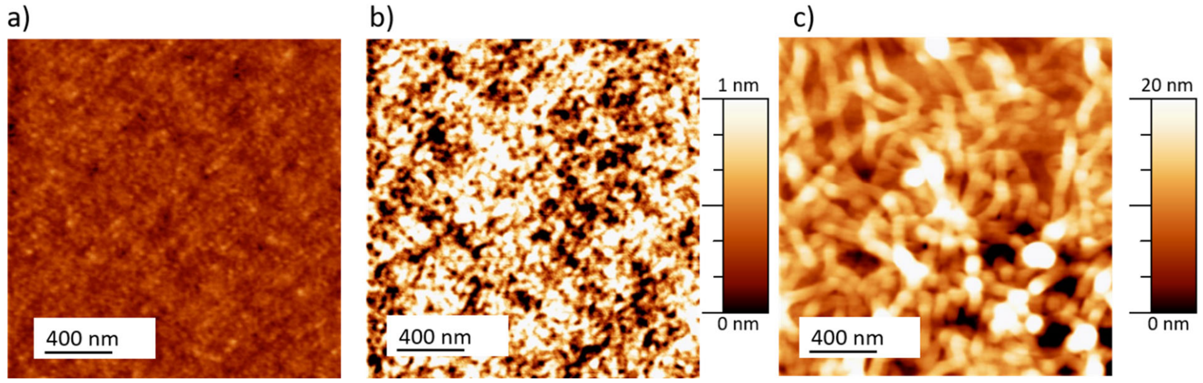

Figure S12: AFM height images of the pristine a) PMMA, b) PMMA-*b*-P3HT and c) P3HT thin films.

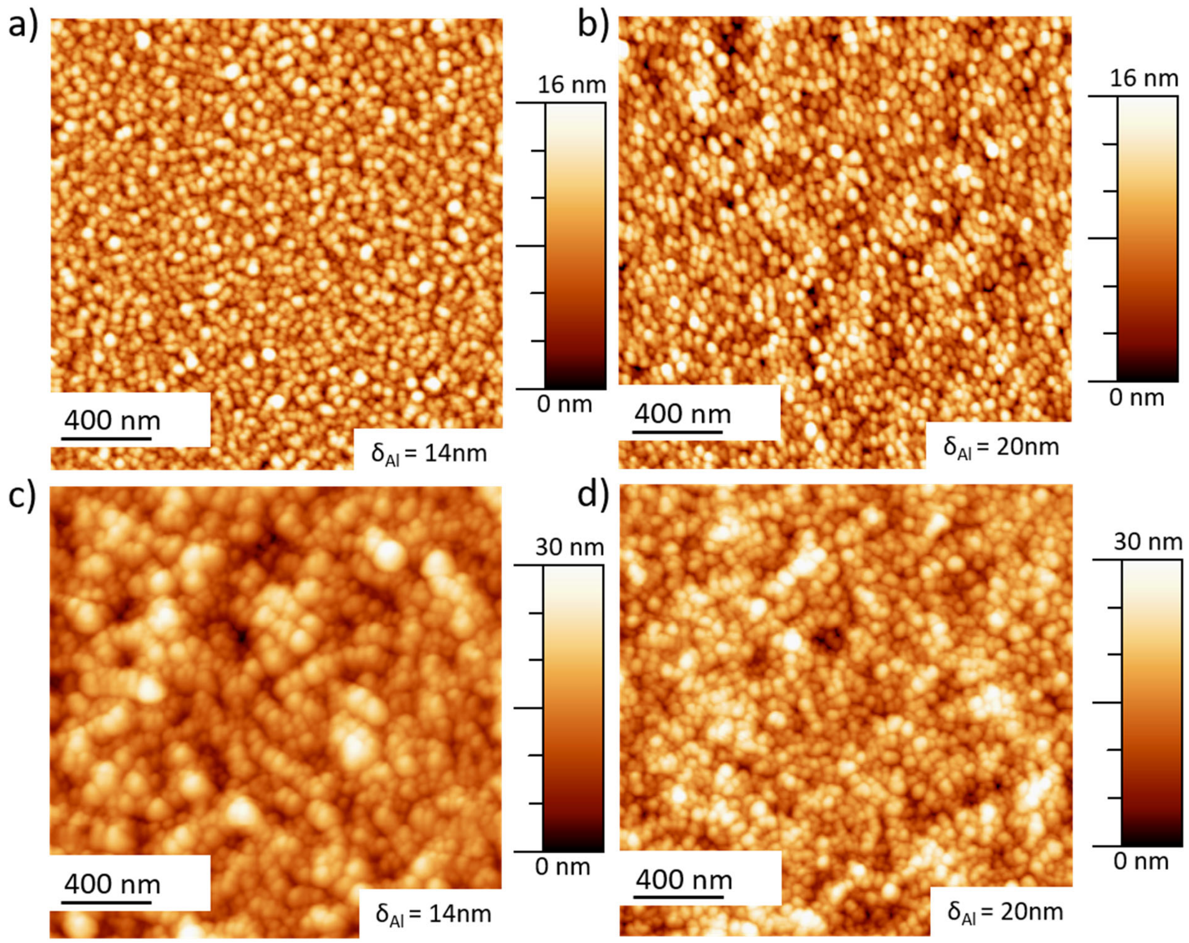

Figure S13: AFM topography of a,b) PMMA and c,d) P3HT with a,c) δ_{Al} = 14 nm and b,d) 20 nm.

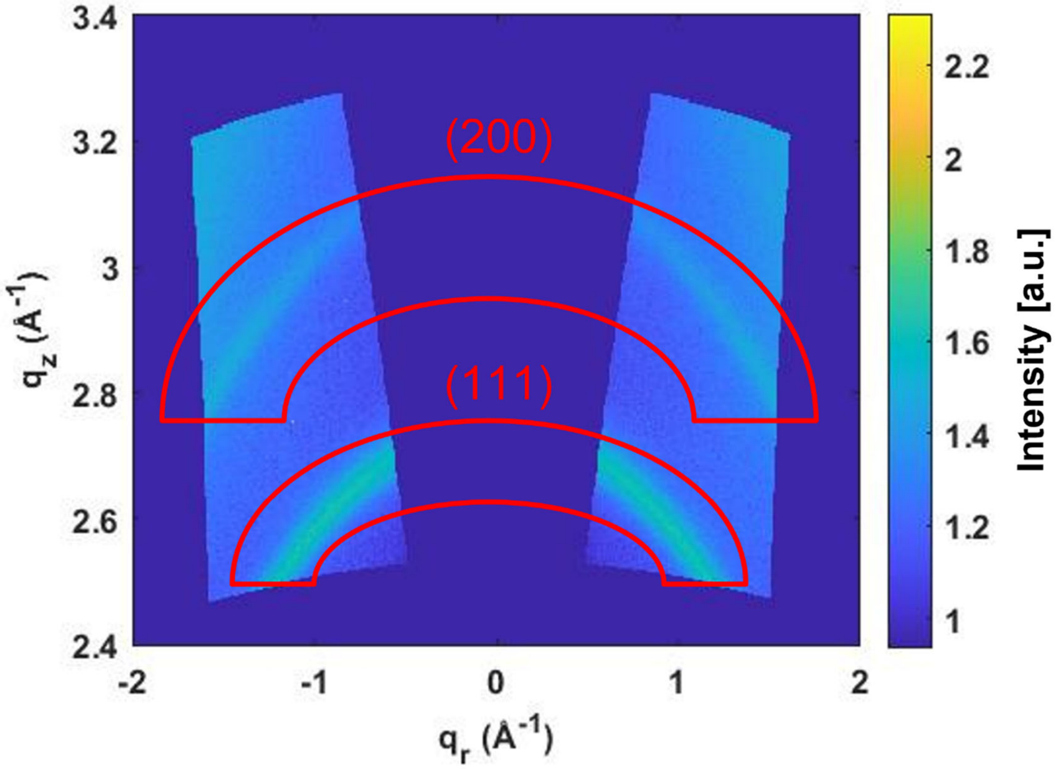

Figure S14: Reshaped 2D GIWAXS data (using GIXSGUI)[8] of PMMA-*b*-P3HT after sputter deposition of δ_{Al} = 20 nm. The (111) and (200) planes of metallic Al are indicated with the red regions.

References:

(1) Schwartzkopf, M.; Hinz, A.; Polonskyi, O.; Strunskus, T.; Löhrer, F. C.; Körstgens, V.; Müller-Buschbaum, P.; Faupel, F.; Roth, S. V. Role of Sputter Deposition Rate in Tailoring Nanogranular Gold Structures on Polymer Surfaces. *ACS Appl. Mater. Interfaces* **2017**, *9*, 5629–5637.

(2) Horcas, I.; Fernández, R.; Gómez-Rodríguez, J. M.; Colchero, J.; Gómez-Herrero, J.; Baro, A. M. WSXM: A Software for Scanning Probe Microscopy and a Tool for Nanotechnology. *Rev. Sci. Instrum.* **2007**, *78*, 013705.

(3) Nečas, D.; Klapetek, P. Gwyddion: An Open-Source Software for SPM Data Analysis. *Cent. Eur. J. Phys.* **2012**, *10*, 181–188.

(4) Buffet, A.; Rothkirch, A.; Döhrmann, R.; Körstgens, V.; Abul Kashem, M. M.; Perlich, J.; Herzog, G.; Schwartzkopf, M.; Gehrke, R.; Müller-Buschbaum, P.; Roth, S. V. P03, the Microfocus and Nanofocus X-Ray Scattering (MiNaXS) Beamline of the PETRA III Storage Ring: The Microfocus Endstation. *J. Synchrotron Radiat.* **2012**, *19*, 647–653.

(5) Benecke, G.; Wagermaier, W.; Li, C.; Schwartzkopf, M.; Flucke, G.; Hoerth, R.; Zizak, I.; Burghammer, M.; Metwalli, E.; Müller-Buschbaum, P.; Trebbin, M.; Förster, S.; Paris, O.; Roth, S. V.; Fratzl, P. A Customizable Software for Fast Reduction and Analysis of Large X-Ray Scattering Data Sets: Applications of the New DPDAK Package to Small-Angle X-Ray Scattering and Grazing-Incidence Small-Angle X-Ray Scattering. *J. Appl. Crystallogr.* **2014**, *47*, 1797–1803.

(6) Schwartzkopf, M.; Röhlsberger, R.; Gehrke, R.; Gehrke, R.; Stribeck, N.; Roth, S. V. From Atoms to Layers : In Situ Gold Cluster Growth Kinetics during Sputter Deposition. *Nanoscale* **2013**, *5*, 5053–5062.

(7) Gensch, M.; Schwartzkopf, M.; Brett, C. J.; Schaper, S. J.; Kreuzer, L. P.; Li, N.; Chen, W.; Liang, S.; Drewes, J.; Polonskyi, O.; Strunskus, T.; Faupel, F.; Müller-Buschbaum, P.; Roth, S. V. Selective Silver Nanocluster Metallization on Conjugated Diblock Copolymer Templates for Sensing and Photovoltaic Applications. *ACS Appl. Nano Mater.* **2021**, *4*, 4245–4255.

(8) Jiang, Z. GIXSGUI: A MATLAB Toolbox for Grazing-Incidence X-Ray Scattering Data Visualization and Reduction, and Indexing of Buried Three-Dimensional Periodic Nanostructured Films. *J. Appl. Crystallogr.* **2015**, *48*, 917–926.
